# Identification and characterisation of transient receptor potential melastatin 2 and CD38 channels on natural killer cells using the novel application of flow cytometry

**DOI:** 10.1186/s12865-019-0293-0

**Published:** 2019-05-10

**Authors:** Cassandra Balinas, Helene Cabanas, Donald Staines, Sonya Marshall-Gradisnik

**Affiliations:** 1 School of Medical Science, Griffith University, Gold Coast, QLD Australia; 2 The National Centre for Neuroimmunology and Emerging Diseases, Menzies Health Institute Queensland, Griffith University, Gold Coast, Southport, QLD 4222 Australia

**Keywords:** Antibody, Flow cytometry, Natural killer cells, Transient receptor potential Melastatin 2

## Abstract

**Background:**

Natural Killer (NK) cells are effector lymphocytes of the innate immune system and are subclassed into CD56^Bright^CD16^Dim/−^ and CD56^Dim^CD16^+^ NK cells. Intracellular calcium (Ca^2+^) is fundamental to regulate a number of intracellular signalling pathways and functions in NK cells, which are essential in mediating their natural cytotoxic function. Transient receptor potential melastatin 2 (TRPM2) is a Ca^2+^-permeable non-selective cation channel that possesses a critical role in calcium-dependent cell signalling to maintain cellular homeostasis. TRPM2 and CD38 protein surface expression has yet to be determined on NK cells using flow cytometry. Characterisation of TRPM2 has been previously identified by in vivo models, primarily using methods such as genetic remodification, immunohistochemistry and whole cell electrophysiology. The aim of this study was to develop an in vitro methodology to characterise TRPM2 and CD38 surface expression on NK cell subsets using an antibody that has not been previously applied using flow cytometry.

**Results:**

At 2 h/1 h, TRPM2 (Fig. 2 A, B, *p < 0.05*) and TRPM2/CD38 (Fig. 3A, B, *p < 0.05*) surface expression significantly increased between 1:300 and 1:50 at 2 h/1 h. TRPM2/CD38 surface expression furthermore increased between 1:100 and 1:50 at 2 h/1 h (Fig. 3A, *p < 0.05*). Interestingly, TRPM2/CD38 surface expression significantly decreased from 1:50 to 1:5 on CD56^Bright^CD16^Dim/−^ NK cells. These consistent findings highlight that 1:50 is the optimal antibody dilution and threshold to measure TRPM2 and TRPM2/CD38 surface expression on NK subsets. 2 h/1 h was determined as the optimal incubation period to ensure a sufficient timeframe for maximal antibody binding and surface expression.

**Conclusion:**

For the first time, we describe an in vitro methodology to characterise TRPM2 and CD38 surface expression on NK cells in healthy participants. Finally, using an antibody that has not been previously applied in flow cytometry, we determined an antibody concentration and incubation time that is robust, rapid and sensitive for the application of flow cytometry.

**Electronic supplementary material:**

The online version of this article (10.1186/s12865-019-0293-0) contains supplementary material, which is available to authorized users.

## Background

Natural Killer (NK) cells are effector lymphocytes of the innate immune system found in peripheral blood, bone marrow, spleen, and lymph nodes. In peripheral blood, NK cells represent 15% of lymphocytes and are phenotypically distinguished by the surface expression of CD56 (neural cell-adhesion molecule) and CD16 (FcγIII receptor, the low affinity receptor of IgG) receptors. Thereby, NK cells are subclassed into CD56^Bright^CD16^Dim/−^ and CD56^Dim^CD16^+^ NK cells which represent respectively 10 and 90% of NK cells in peripheral blood [1]. NK cells have diverse biological functions, which include recognizing and killing virally infected or transformed cells. The former NK population is primarily involved in immunosurveillance and cytokine production, whereas the latter are cytotoxic and kill infected, tumour or ‘missing self’ cells [2]. Intracellular calcium (Ca^2+^) mobilisation is required to regulate a number of intracellular signalling pathways in NK cells, such as the antibody dependent cellular cytotoxicity (ADCC) or mitogen-activated protein kinase pathway, which are essential for the development of immune synapse formation, cytokine production and cytotoxic activity [1]. Intracellular Ca^2+^ is also required for the target cell adhesion, granule polarization and degranulation, all of which are necessary for NK cells to mediate their natural cytotoxicity [1, 3].

Transient receptor potential melastatin 2 (TRPM2) is a Ca^2+^-permeable nonselective cation channel that is characterised with a unique C-terminal ADP-ribose (ADPR) pyrophosphate domain [4]. TRPM2 is synergistically activated by intracellular ADPR and Ca^2+^ within the plasma membrane and/or lysosomal compartments. Binding of ADPR to TRPM2 opens the channel and allows the permeation of sodium (Na^2+^), potassium (K^+^) and Ca^2+^ into the cell and hydrolysis of ADPR to ribose 5-phosphate and adenosine monophosphate (AMP) [5]. Previous investigations have shown that TRPM2 mediates a novel anti-tumour mechanism in NK cells in synergy with CD38, a multifunctional ectoenzyme using Nicotinamide adenine dinucleotide (NAD^+^) as a substrate to catalyse the production of ADPR, cyclic ADPR (cADPR) and Nicotinic acid adenine dinucleotide phosphate (NAADP) [6]. Rah et al. (2016) demonstrated that CD38 facilitates the production of ADPR, which in turn mobilizes intracellular Ca^2+^ and can activate TRPM2 resulting in cytolytic degranulation and antitumor activity of NK cells [6].

Investigation of TRP ion channel expression on lymphocytes has been quite limited due to methodology difficulties as TRP channels are relatively low in abundance and there is limited availability of specific and high-affinity antibodies. Characterisation of TRPM2 has been predominantly investigated with in vivo models accompanied by genetic remodification [6–16], western blot [6, 13, 17], immunohistochemistry [6, 12, 15], polymerase chain reaction [10–13, 15–18], and whole cell-electrophysiology [8–11, 13, 18] methods. In vitro investigations of TRPM2 have furthermore been examined on cell lines [10, 17–20], neurons [21, 22], and immune cells [10–13, 16].

TRPM3 surface expression on CD56^Bright^CD16^Dim/−^ and CD56^Dim^CD16^+^ NK cells has been identified on healthy participants by flow cytometry [23, 24]. Flow cytometry has been the preferred technology for determining and quantifying homogenous cell subsets [25] due to its single-cell levelled analysis for multiple characteristics, such as cellular features, organelles, and structural components [25]. This sensitive and specific feature enables prompt and accurate quantification, analytical precision, superior throughput, and reproducibility [26], all of which are advantageous for unique and rare cell populations, such as NK cells. Current flow cytometer technologies can detect up to eighteen colours in one flow assay. Thus, the scientific prospects not only lie in biomedical research, but also for clinical applications of diagnostic value [25].

Currently, there are no in vitro models that have characterised endogenous TRPM2 and CD38 surface expression on human NK cells. Thus, the aim of this present study was to develop a methodology to characterise TRPM2 and CD38 surface expression on human NK cells using flow cytometry. This investigation may facilitate a better understanding of the role of TRPM2 and CD38 in disease pathology involving immune cells such as NK cells.

## Results

### Immunophenotype of TRPM2 and CD38 receptors on NK cell subsets by flow cytometry

CD3^−^/CD56^+^ NK cells were sorted into CD56^Dim^CD16^/+^ and CD56^Bright^CD16^Dim/−^ NK cell subsets using CD56 (Pe-Cy7) and CD16 (BV650). Five antibody controls were performed to determine an individualised positive TRPM2 and TRPM2/CD38 gate for each participant. Antibody controls included an unstained tube (unlabelled NK cells); secondary tube (conjugated secondary antibody FITC); and a FMO tube (CD3, CD56, CD16 and CD38). (b) Normal rabbit serum was used at comparable dilutions as the primary TRPM2 antibody to measure TRPM2 and TRPM2/CD38 surface expression on NK subsets. (c) Normalised TRPM2 and TRPM2/CD38 surface expression was calculated by compensating the percentage of fluorescence spill over into the B525_50 (TRPM2) and V525_50 (CD38) detectors from the TRPM2 antibody stained tube on both NK subsets.

### Flow cytometry gating strategy of TRPM2 and CD38 using primary TRPM2 antibody (1:50) at 2 h/1 h

**Fig. 1:**
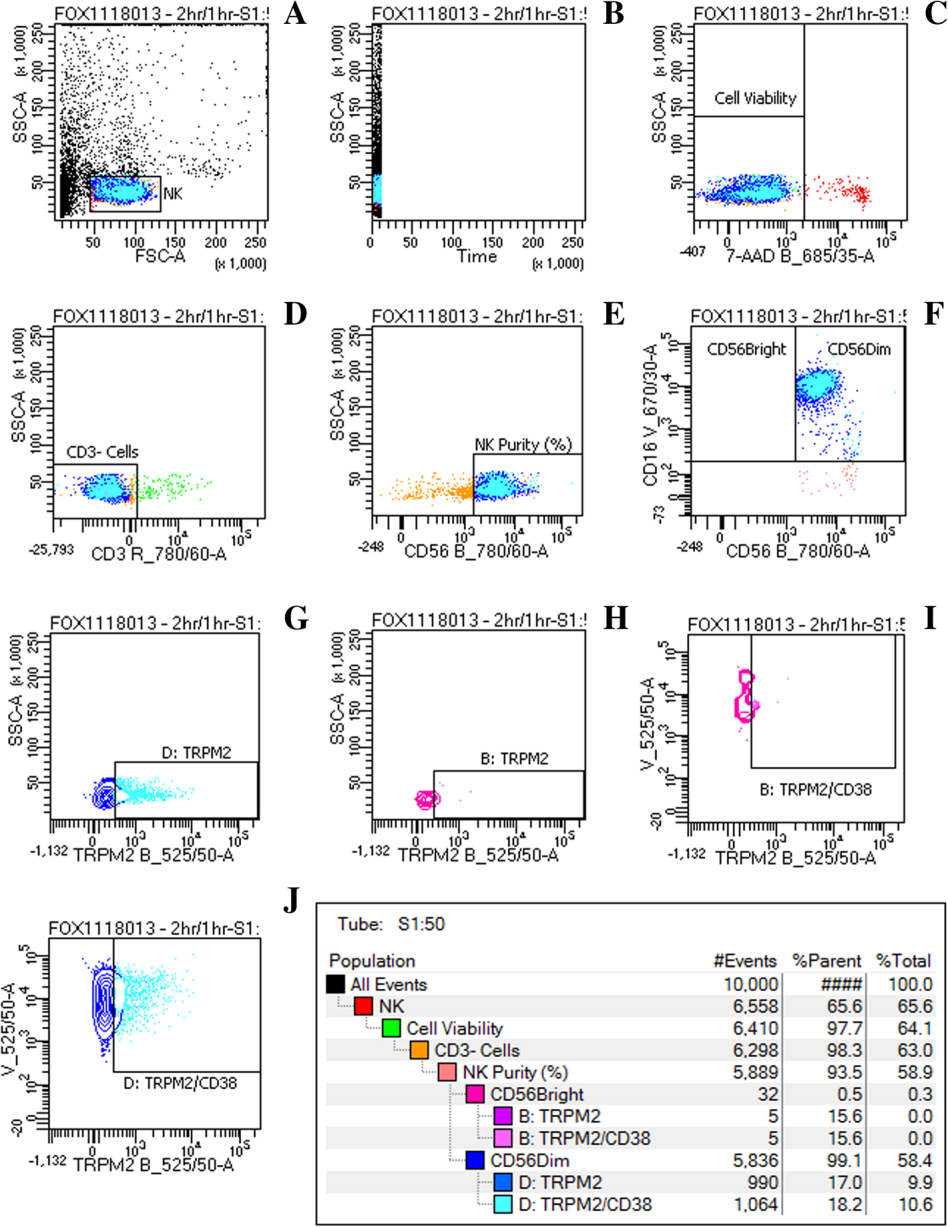
Flow cytometry gating strategy of TRPM2 and CD38 using primary TRPM2 antibody (1:50) at 2 h/1 h. **a** Lymphocyte populations were identified using forward and side scatter dot plots. **b** Duration of cell acquirement was measured followed by (**c**) cell viability after 7-AAD staining. **d** CD3^+^ cells were excluded and only (**e**) CD3^-^ lymphocytes were further used to characterise NK cells by CD56. **f** CD3^−^/CD56^+^ NK cells were sorted into CD56^Bright^CD16^Dim/−^ and CD56^Dim^ CD16^+^ NK cell subsets using CD56 and CD16. TRPM2 surface expression was measured on (**g**) CD56^Dim^ CD16^+^ and (**h**) CD56^Bright^CD16^Dim/−^ NK cell subsets. Dual surface expression of TRPM2 and CD38 was furthermore assessed on (**i**) CD56^Bright^CD16^Dim/−^ and (**j**) CD56^Dim^ CD16^+^ NK cell subsets

**(a)** Lymphocyte populations were identified using forward and side scatter dot plots. (**b)** Duration of cell acquirement was measured followed by **(c)** cell viability after 7-AAD staining. **(d)** CD3^+^ cells were excluded and only **(e)** CD3^−^ lymphocytes were further used to characterise NK cells by CD56. **(f)** CD3^−^/CD56^+^ NK cells were sorted into CD56^Bright^CD16^Dim/−^ and CD56^Dim^CD16^+^ NK cell subsets using CD56 and CD16. TRPM2 surface expression was measured on **(g)** CD56^Dim^CD16^+^ and **(h)** CD56^Bright^ CD16^Dim/−^ NK cell subsets. Dual surface expression of TRPM2 and CD38 was furthermore assessed on **(i)** CD56^Bright^CD16^Dim/−^ and **(j)** CD56^Dim^CD16^+^ NK cell subsets (Fig. 1).

### TRPM2 surface expression on natural killer cell subsets in healthy participants

**Fig. 2:**
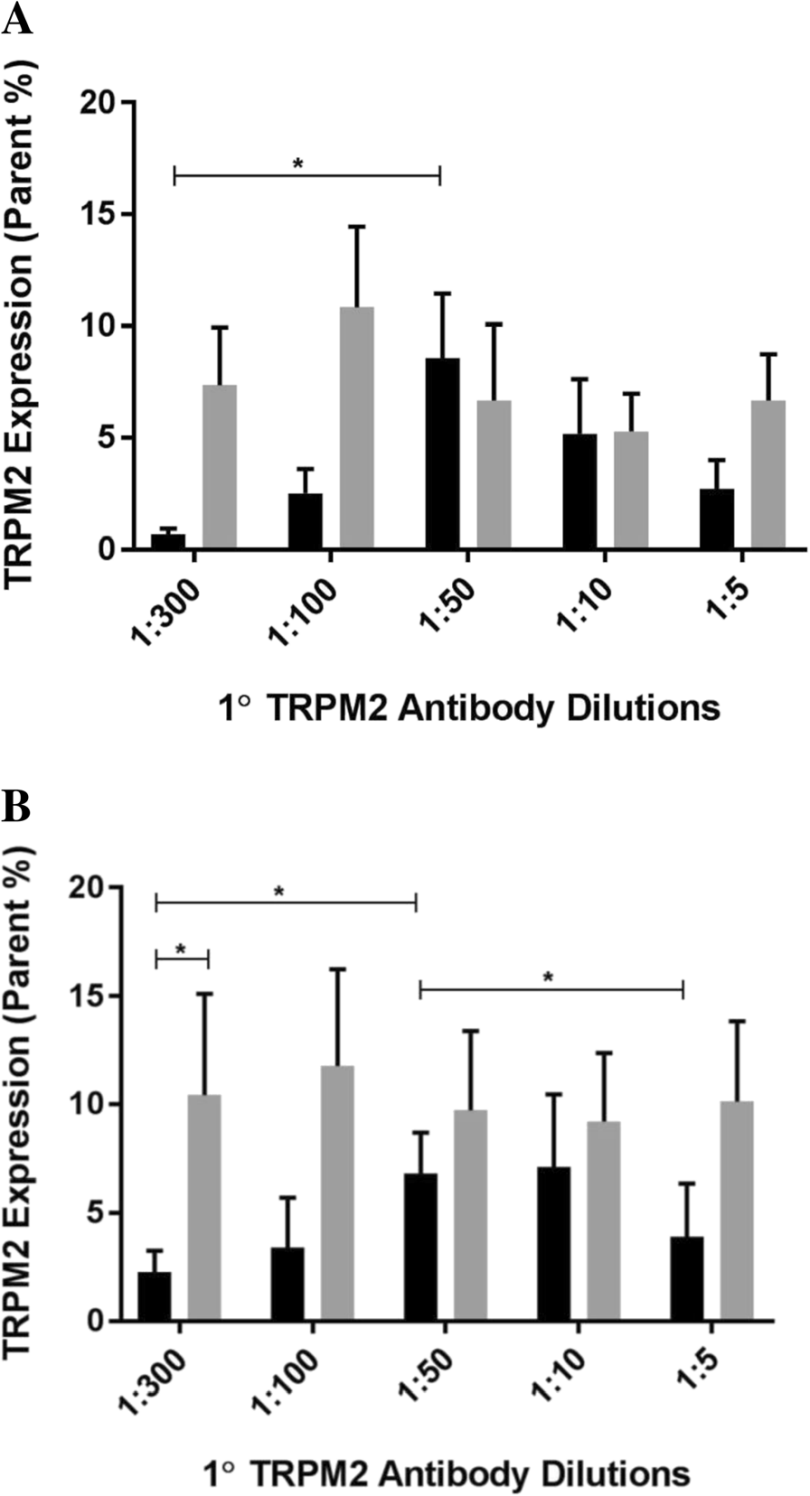
TRPM2 surface expression on Natural Killer cell subsets in healthy participants. TRPM2 surface expression was measured on (**a**) CD56^Dim^CD16^+^ and (**b**) CD56^Bright^CD16^Dim/−^ NK cells by flow cytometry. TRPM2 surface expression was comparatively measured at 2 h/1 h [black] and 1 h/30 min [grey] following 1:300, 1:100, 1:50, 1:10 and 1:5 primary TRPM2 antibody dilutions. Representative flow cytometry dot plots of each respective dilution are presented in Additional file 4: Figure S4, Additional file 5: Figure S5, Additional file 6: Figure S6, Additional file 7: Figure S7, Additional file 8: Figure S8, Additional file 9: Figure S9, Additional file 10: Figure S10, Additional file 11: Figure S11, Additional file 12: Figure S12, Additional file 13: Figure S13 and Additional file 17: Figure S17, Additional file 18: Figure S18, Additional file 19: Figure S19, Additional file 20: Figure S20, Additional file 21: Figure S21, Additional file 22: Figure S22, Additional file 23: Figure S23, Additional file 24: Figure S24, Additional file 25: Figure S25, Additional file 26: Figure S26. Bar graphs report the means ± SEM. Mann Whittney U test and Kruskal Wallis H test were performed. * *p < 0.05*. *1°* primary, *TRPM2*, Transient Receptor Potential Melastatin 2

To quantify the surface expression on different NK cell subsets, CD56^Bright^CD16^Dim/−^ and CD56^Dim^CD16^+^ NK subsets were acquired following CD56 and CD16 antibody staining. On CD56^Dim^CD16^+^ (Fig. 2a, *p < 0.05*) and CD56^Bright^CD16^Dim/−^ (Fig. 2b, *p < 0.05*) NK subsets, TRPM2 surface expression significantly increased between 1:300 and 1:50 at 2 h/1 h. On the CD56^Bright^CD16^Dim/−^ subset, TRPM2 surface expression significantly decreased from 1:50 to 1:5 at 2 h/1 h (Fig. 2b, *p < 0.05*). Moreover, a significant increase in TRPM2 surface expression was observed between incubation periods within the 1:300 dilution (Fig. 2b, *p < 0.05*).

### Dual identification of TRPM2 and CD38 surface expression on natural killer cell subsets

**Fig. 3:**
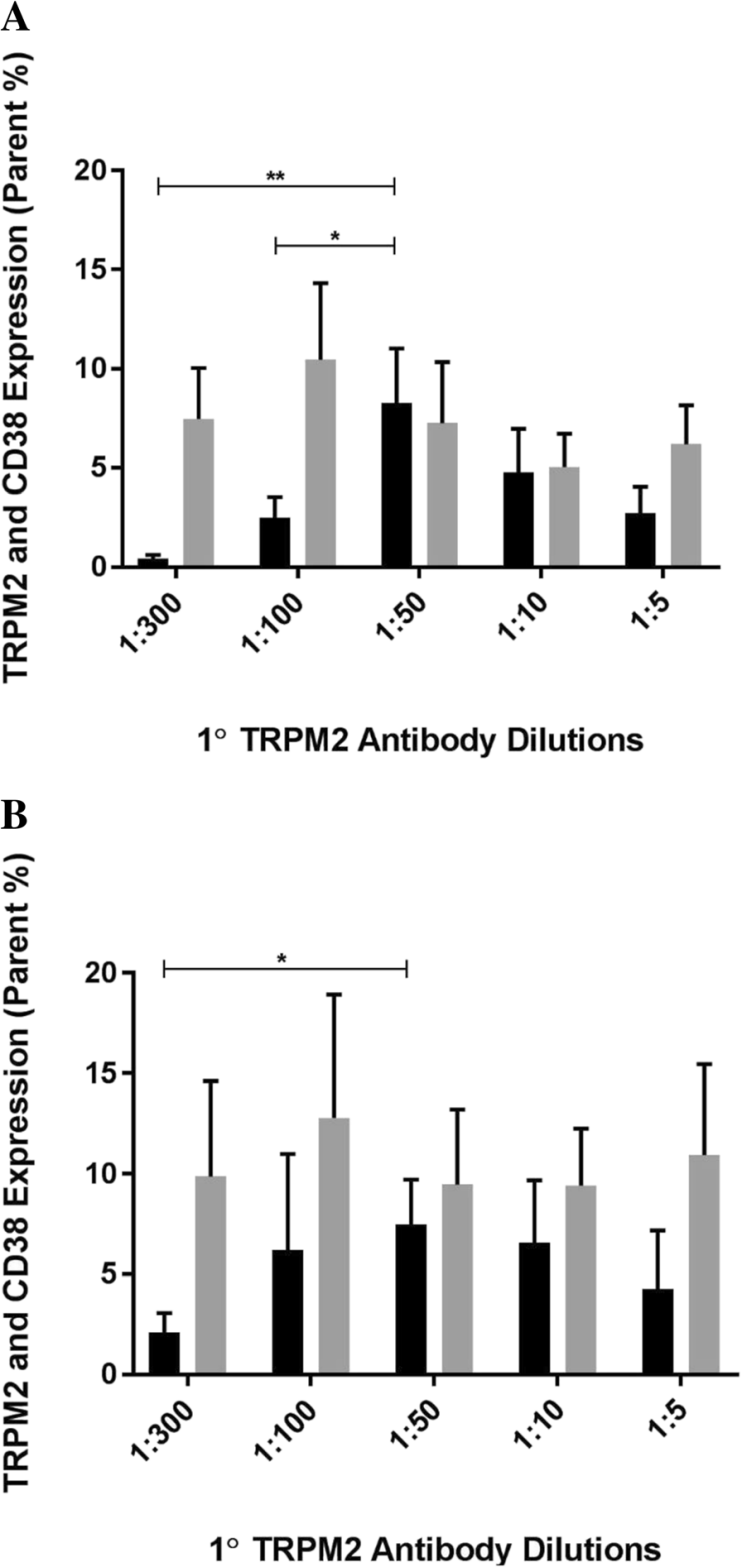
Dual identification of TRPM2 and CD38 surface expression on Natural Killer cell subsets. TRPM2/CD38 surface expression was measured on (**a**) CD56^Dim^CD16^+^ and (**b**) CD56^Bright^CD16^Dim/−^ NK cells by flow cytometry. TRPM2/CD38 surface expression was comparatively measured at 2 h/1 h [black] and 1 h/30 min [grey] following 1:300, 1:100, 1:50, 1:10 and 1:5 primary TRPM2 antibody dilutions. Representative flow cytometry dot plots of each respective dilution are presented in Additional file 4: Figure S4, Additional file 5: Figure S5, Additional file 6: Figure S6, Additional file 7: Figure S7, Additional file 8: Figure S8, Additional file 9: Figure S9, Additional file 10: Figure S10, Additional file 11: Figure S11, Additional file 12: Figure S12, Additional file 13: Figure S13 and Additional file 17: Figure S17, Additional file 18: Figure S18, Additional file 19: Figure S19, Additional file 20: Figure S20, Additional file 21: Figure S21, Additional file 22: Figure S22, Additional file 23: Figure S23, Additional file 24: Figure S24, Additional file 25: Figure S25, Additional file 26: Figure S26. Bar graphs report the means ± SEM. Mann Whittney U test and Kruskal Wallis H test were performed. * *p < 0.05*, ** p < 0.01. 1° primary, *TRPM2*, Transient Receptor Potential Melastatin 2

To further explore the co-expression of TRPM2 and CD38, isolated NK subsets were additionally stained with CD38. On CD56^Dim^CD16^+^ (Fig. 3a, *p < 0.05*) and CD56^Bright^CD16^Dim/−^ (Fig. 3b, *p < 0.05*) NK cell subsets, TRPM2 and CD38 surface expression significantly increased between 1:300 and 1:50 at 2 h/1 h. Additionally, a significant increase in TRPM2 and CD38 surface expression was observed between 1:100 and 1:50 on CD56^Bright^CD16^Dim/−^ (Fig. 3b, *p < 0.05*) at 2 h/1 h.

## Discussion

This investigation reports, for the first time, the identification of TRPM2 and CD38 surface expression on human NK cell subsets in healthy participants. This paper is also the first to develop a methodology that quantifies TRPM2 and CD38 surface expression with an antibody that has not been previously applied using flow cytometry. This novel method may have significant implications for analysing TRPM2 and CD38 surface expression in vitro and may facilitate a better understanding of the role of TRPM2 and CD38 in disease pathology involving immune cells such as NK cells.

In order to characterise TRPM2 surface expression, an extracellular TRPM2 antibody was preferred to prevent non-specific binding. The predominant clonality available on the market is polyclonal intracellular TRPM2 antibodies. Intracellular TRPM2 ion channels were not investigated as cell fixation and permeabilisation provides access to intracellular antigens. As TRPM2 is also localised on intracellular compartments, such as the endoplasmic reticulum and lysosome, cell permeabilisation can enable non-specific binding and activation of these intracellular TRPM2 channels, which potentially can mediate a number of downstream signalling pathways, such as Ca^2+^ influx (15). Thus, a rabbit IgG polyclonal extracellular TRPM2 antibody (Thermo Fisher Scientific, USA*,* OST00112W) was chosen due to its ready availability and extracellular binding, specifically to the third extracellular loop of the human TRPM2 receptor.

A total of eight healthy Australian participants were age (27.50 ± 8.08) and sex-matched (Table 1). No significant differences were reported for full blood count parameters between participants (Table 2). As this project was the first to use an antibody that has only been used for western blot and immunohistochemistry, the recommended dilution series (1:300) by the manufactures’ instructions was used as a baseline. With this reference, four additional primary TRPM2 antibody dilutions (1:100, 1:50, 1:10 and 1:5) were investigated to determine the optimal primary TRPM2 antibody concentration. Additionally, two incubation periods (1 h – primary TRPM2 antibody/30 min – secondary conjugated TRPM2 antibody) and (2 h/1 h) were tested to determine the optimal incubation time for TRPM2 and TRPM2/CD38 surface binding and expression (Additional file 4: Figure S4, Additional file 5: Figure S5, Additional file 6: Figure S6, Additional file 7: Figure S7, Additional file 8: Figure S8, Additional file 9: Figure S9, Additional file 10: Figure S10, Additional file 11: Figure S11, Additional file 12: Figure S12, Additional file 13: Figure S13, Additional file 17: Figure S17, Additional file 18: Figure S18, Additional file 19: Figure S19, Additional file 20: Figure S20, Additional file 21: Figure S21, Additional file 22: Figure S22, Additional file 23: Figure S23, Additional file 24: Figure S24, Additional file 25: Figure S25, Additional file 26: Figure S26).

**Table 1:** Demographic results of healthy participants

Parameters	Healthy participants	*p* value
Age (years)	27.50 ± 8.08	0.349
Gender		
Male (*n* = 4)	50%	
Female (n = 4)	50%	

Participant Demographics

A total of eight healthy Australian participants were included for this present study and there were no significant differences in age and gender between healthy participants (Table 1)

**Table 2:** Full blood count parameters of healthy participants

Parameters	Healthy Male Participants	Healthy Female Participants	*P* value
White Cell count (× 10^9^/L)	5.57 ± 0.41	6.14 ± 0.35	0.310
Neutrophils (× 10^9^/L)	3.27 ± 0.30	3.76 ± 0.20	0.280
Lymphocytes (× 10^9^/L)	1.58 ± 0.14	1.55 ± 0.29	0.258
Monocytes (× 10^9^/L)	0.43 ± 0.03	0.51 ± 0.04	0.314
Eosinophils (× 10^9^/L)	0.24 ± 0.05	1.15 ± 0.10	0.628
Basophils (× 10^9^/L)	0.06 ± 0.01	0.08 ± 0.20	0.489
Platelets (× 10^9^/L)	2.48 ± 0.72	2.32 ± 1.54	0.08
Haemoglobin (× 10^9^/L)	142.10 ± 3.99	148.56 ± 4.50	0.954
Red Cell count (× 10^12^/L)	4.89 ± 0.15	4.90 ± 0.16	0.297
MCV (× 10^9^/L)	88.10 ± 1.75	83.29 ± 1.81	0.554

Full blood count parameters were measured for each healthy participant. All participant results were within the specified reference ranges for each parameter. There were no significant differences between healthy participants for these reporting parameters (Table 2)

**Fig. 4:**
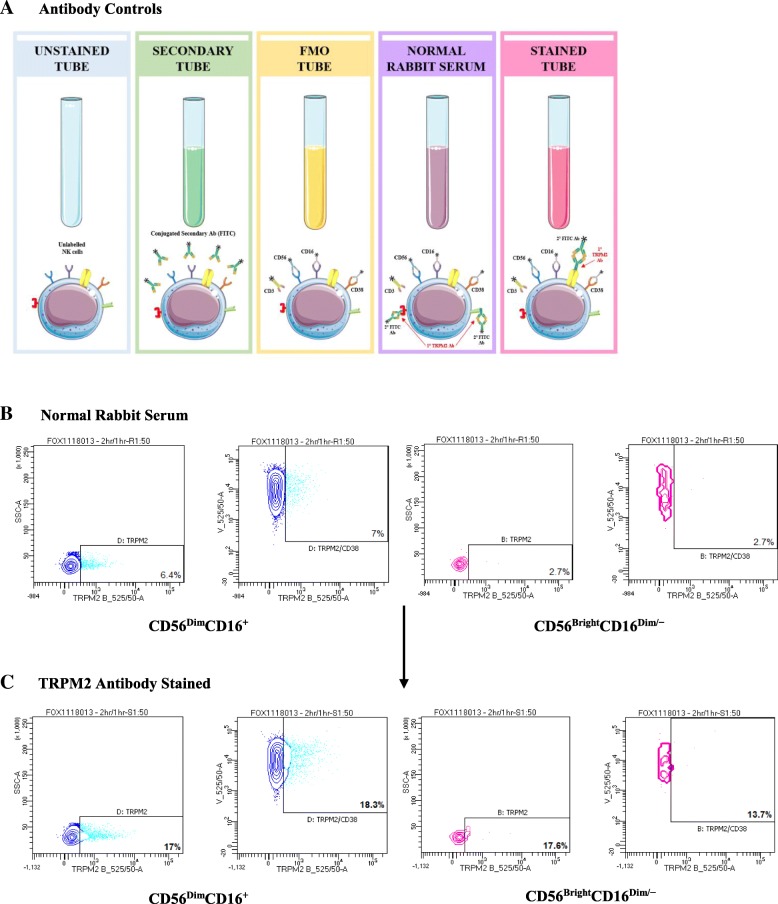
Immunophenotype of TRPM2 and CD38 receptors on NK cell subsets by flow cytometry. (Source: Personal collection) **a** Five antibody controls were performed to determine an individualised positive TRPM2 and TRPM2/CD38 gate for each participant. Antibody controls included an unstained tube (unlabelled NK cells); secondary tube (conjugated secondary antibody FITC); and a FMO tube (CD3, CD56, CD16 and CD38). **b** Normal rabbit serum was used at comparable dilutions as the primary TRPM2 antibody to measure TRPM2 and TRPM2/CD38 surface expression on NK subsets. **c** Normalised TRPM2 and TRPM2/CD38 surface expression was calculated by compensating the percentage of fluorescence spill over into the B525_50 (TRPM2) and V525_50 (CD38) detectors from the TRPM2 antibody stained tube on both NK subsets

One limitation of the primary TRPM2 antibody was the absence of a determined antibody concentration. According to Thermo Fisher Scientific, “antibody concentrations in ascites fluid, culture supernatant and serum are not determined due to various proteins in serum which makes it impossible to acquire an accurate concentration of a specific antibody”. Due to the absence of a determined antibody concentration, a TRPM2 isotype control could not be performed. However, as the primary TRPM2 antibody contains rabbit serum, normal rabbit serum (Thermo Fisher Scientific, USA, 01–6101) was used at comparable dilutions as the primary TRPM2 antibody. This negative control was used to distinguish any non-specific binding, as well as determine an individual positive TRPM2 and TRPM2/CD38 gate for each participant (Fig. 4b, c). Additionally, an unstained tube; a secondary tube; and a FMO control (Fig. 4a) were performed for each participant to compensate any potential fluorescence spill over (Additional file 1: Figure S1, Additional file 2: Figure S2, Additional file 3: Figure S3, Additional file 14: Figure S14, Additional file 15: Figure S15, Additional file 16: Figure S16).

On both NK subsets, a consistent pattern was observed for TRPM2 and dual surface expression with CD38. At 2 h/1 h, TRPM2 (Fig. 2a, b, *p < 0.05*) and TRPM2/CD38 (Fig. 3a, b, *p < 0.05*) surface expression significantly increased between 1:300 and 1:50 at 2 h/1 h. Additionally, a significant increase in TRPM2/CD38 expression was also observed on CD56^Dim^CD16^+^ NK cells between 1:100 and 1:50 at 2 h/1 h (Fig. 3a, *p < 0.05*). These results indicate that 1:50 may be the optimal antibody concentration to measure TRPM2 and TRPM2/CD38 surface expression on NK cells.

The specificity of the primary TRPM2 antibody was investigated by measuring the dual surface expression of co-markers, TRPM2 and CD38, on CD56^Bright^CD16^Dim/−^ and CD56^Dim^CD16^+^ NK cells. Given comparable results were observed with (Fig. 3a, b, *p < 0.05*) and without CD38 expression (Fig. 2a, b, *p < 0.05*), these findings validate the specificity of the TRPM2 antibody for accurate and consistent measurement of TRPM2 surface expression.

Interestingly, a normal distribution curve was observed on both NK subsets for TRPM2 and dual expression with CD38 at 2 h/1 h. Comparatively, receptor surface expression remained relatively constant at 1 h/30 min on both NK subsets. This observation supported the significant decrease in TRPM2/CD38 surface expression from 1:50 to 1:5 on CD56^Bright^CD16^Dim/−^ NK cells (Fig. 3a, *p < 0.05*). Importantly, this result demonstrates an inverse relationship between antibody concentration and receptor expression and highlights 1:50 as the threshold antibody dilution for TRPM2 (Fig. 1).

In contrast there was a significant increase in TRPM2 surface expression with 1:300 at 1 h/30 min (Fig. 2b, *p < 0.05*), but not with dual expression with CD38 (Fig. 2b, *p < 0.05*). This sole result revealed a difference in receptor surface expression between incubation periods. As CD56^Bright^CD16^Dim/−^ NK cells are less abundant than the CD56^Dim^CD16^+^ subset, the percentage of receptor expression increases with limited cells detected. Moreover, the percentage of receptor expression increases for rarer channels. Given TRP ion channels are relatively scarce, particularly on lymphocytes, a longer incubation time is required to ensure optimal binding and subsequent surface expression. Moreover, the consistent pattern with the 1:50 TRPM2 dilution on both NK subsets justified 2 h/1 h as the optimal incubation period to ensure a sufficient timeframe for maximal antibody binding and surface expression.

Despite tested applications for western blot and immunohistochemistry assays, no additional studies have published the use of the OST00112W TRPM2 antibody. Future directions include the examination of TRPM2 and CD38 channels on additional lymphocytes, as well as investigate the manufacturer’s tested applications to further assess antibody specificity.

## Conclusion

This novel methodology is the first to identify and characterise TRPM2 and TRPM2/CD38 surface expression on human NK cells in healthy participants. This pilot investigation is also the first to use a TRPM2 antibody that has not been previously applied in flow cytometry, as well as determine the optimal primary TRPM2 antibody dilution and incubation time. This method provides an in vitro alternative using flow cytometry to characterise TRPM2 in a rapid, robust and sensitive fashion. This pilot investigation provides insight for possible improvement in antibody design to facilitate a more accurate assessment of TRPM2 and CD38 surface expression.

## Methods

### Study participants

From 150 screened Australian participants, ten healthy participants were selected for this pilot investigation. Two participants were excluded due to outlier values during data analysis. Participants were sourced from the National Centre of Neuroimmunology and Emerging Diseases (NCNED) database for Chronic Fatigue Syndrome/Myalgic Encephalomyelitis (CFS/ME) between November and December 2018. Participants were excluded if they were pregnant or breastfeeding, or reported a previous history of smoking, alcohol abuse or chronic illness (for example, autoimmune diseases, cardiac diseases and primary psychological disorders). Participants donated 85 ml of whole blood in ethylendiaminetetraacetic acid (EDTA) tubes between 8:30 am and 10:00 am on the Gold Coast. All healthy participants provided written consent and the study was approved by the Griffith University Human Research Ethics Committee (HREC/15/QGC/63).

### Peripheral blood mononuclear cell isolation and natural killer cell isolation

Peripheral blood mononuclear cells (PBMCs) were isolated from whole blood by centrifugation over a density gradient medium (Ficoll-Paque Premium; GE Healthcare, Uppsala, Sweden) to separate granulocytes as previously described [27, 28]. PBMCs were stained with trypan blue stain (Invitrogen, Carlsbad, CA) to determine total cell count and cell viability and adjusted to a final concentration of 5 × 10^7^ cells/ml. NK cells were isolated from PBMCs using an EasySep Negative Human NK Cell Isolation Kit (Stemcell Technologies, Vancouver, BC, Canada) as previously described [27, 28].

### TRPM2 Immunophenotyping assay

Following magnetic NK cell isolation, NK cells were stained with trypan blue stain (Invitrogen, Carlsbad, CA) to determine live cell count and cell viability and adjusted to a final concentration of 1.04 × 10^5^ cells/ml. NK cells were incubated with an Fc receptor Blocking reagent (Miltenyi Biotech, Bergisch Gladbach. Germany) for 10 min at 4 °C prior to antibody staining. NK cells were incubated with primary fluorochrome labelled antibodies (CD3-APCH7 [0.5μg/5ul], CD56-PeCy7 [0.25μg/5ul], CD16-BV650 [0.25μg/5ul], and CD38-BV480 [1μg/5ul]) purchased from BD Bioscience), in addition to an unconjugated rabbit IgG polyclonal extracellular TRPM2 antibody (Thermo Fisher Scientific, USA, OST00112W). The primary TRPM2 antibody was resuspended in 100 μl of distilled water according to manufacturer’s instructions. Using the recommended dilution (1:300), five dilution series of the primary (1°) TRPM2 antibody were performed (1:300, 1:100, 1:50, 1:10 and 1:5) with an end volume of 100 μl per test. Normal rabbit serum (Thermo Fisher Scientific, USA, 01–6101) was used as a negative control to determine an individualised positive TRPM2 gate for each participant. Comparable dilutions (1:300, 1:100, 1:50, 1:10 and 1:5) to the primary TRPM2 antibody were applied. Additionally, an unstained tube (unlabelled NK cells); a secondary tube (secondary antibody only); and a Fluorescence Minus One (FMO) (CD56, CD3, CD16 and CD38) control were performed (Additional file 1: Figure S1, Additional file 2: Figure S2, Additional file 3: Figure S3, Additional file 14: Figure S14, Additional file 15: Figure S15, Additional file 16: Figure S16). Normalised TRPM2 and CD38 surface expression was calculated by compensating the percentage of fluorescence spill over into the B525/50 (TRPM2) and V525/50 (CD38) and outlined below:

$$ TRPM2\  Surface\ Expression= TRPM2\  Stained\ tube\ \left( parent\%\right)- Normal\ Rabbit\ Serum\ \left( parent\%\right) $$

Two incubation periods were performed for the primary and secondary TRPM2 antibodies (1 h and 30 min vs. 2 h and 1 h) at 4 °C in the dark. Labelled cells were washed with stain buffer (BD Biosciences, New Jersey, USA) and centrifuged at 350 g for 5 min. Supernatant was removed and cells were incubated with a secondary Goat F(ab) Anti-Rabbit IgG H&L Fluorescein isothiocyanate (FITC) (1:500, Abcam, UK, ab7050) in 200 μl for 1 h/30 min. Cells were washed and stained with 5 μl of 7-AAD (BD Bioscience, New Jersey, USA) to measure cell viability. Cells were resuspended in 200 μl of stain buffer (BD Bioscience, New Jersey, USA) and acquired at 10,000 events using the LSRFortessa X-20.

### LSR Fortessa X-20 flow cytometry analysis

Lymphocyte populations were identified using forward and side scatter dot plots. Exclusions were CD3^+^ cells and only CD3^−^ lymphocytes were further used to characterise NK cells by CD56. CD3^−^/CD56^+^ NK cells were sorted into CD56^Bright^CD16^Dim/−^ and CD56^Dim^CD16^+^ NK cell subsets using CD56 and CD16. TRPM2 and CD38 surface expression was measured on CD56^Bright^CD16^Dim/−^ and CD56^Dim^CD16^+^ NK cell populations as percentage of parent cells (%).

### Statistical analysis

Pilot data from this investigation were analysed using SPSS version 24 (IBM Corp, Version 24, Armonk, NY, USA) and GraphPad Prism, version 7 (GraphPad Software Inc., Version 7, La Jolla, CA, USA). Shapiro-Wilk normality tests were conducted to determine the distribution of data, in addition to skewness and kurtosis tests to determine data normality. The independent Mann–Whitney U test was performed to determine the statistical significance between groups in TRPM2 parameters on NK cells. Conversely, the Kruskal Wallis H test was used to determine significance in TRPM2 and CD38 surface expression within groups. Significance was set at *p* < 0.05 and the data are presented as mean ± standard error of the mean unless otherwise stated.

## Additional files

Additional file 1: Gating strategy for the analysis of TRPM2 and CD38 surface expression on unlabelled NK cells at 2 h/1 h. (PDF 39 kb)

Additional file 2: Gating strategy for the analysis of TRPM2 and CD38 surface expression on NK subsets using a secondary antibody control at 2 h/1 h. (PDF 40 kb)

Additional file 3: Gating strategy for the analysis of TRPM2 and CD38 surface expression on NK subsets using a fluorescence minus one control at 2 h/1 h. (PDF 42 kb)

Additional file 4: Gating strategy for the analysis of TRPM2 and CD38 surface expression on NK subsets using normal rabbit serum (1:300) at 2 h/1 h. (PDF 43 kb)

Additional file 5: Gating strategy for the analysis of TRPM2 and CD38 surface expression on NK subsets using normal rabbit serum (1:100) at 2 h/1 h. (PDF 44 kb)

Additional file 6: Gating strategy for the analysis of TRPM2 and CD38 surface expression on NK subsets using normal rabbit serum (1:50) at 2 h/1 h. (PDF 44 kb)

Additional file 7: Gating strategy for the analysis of TRPM2 and CD38 surface expression on NK subsets using normal rabbit serum (1:10) at 2 h/1 h. (PDF 44 kb)

Additional file 8: Gating strategy for the analysis of TRPM2 and CD38 surface expression on NK subsets using normal rabbit serum (1:5) at 2 h/1 h. (PDF 44 kb)

Additional file 9: Gating strategy for the analysis of TRPM2 and CD38 surface expression on NK subsets using primary TRPM2 antibody (1:300) at 2 h/1 h. (PDF 43 kb)

Additional file 10: Gating strategy for the analysis of TRPM2 and CD38 surface expression on NK subsets using primary TRPM2 antibody (1:100) at 2 h/1 h. (PDF 44 kb)

Additional file 11: Gating strategy for the analysis of TRPM2 and CD38 surface expression on NK subsets using primary TRPM2 antibody (1:50) at 2 h/1 h. (PDF 44 kb)

Additional file 12: Gating strategy for the analysis of TRPM2 and CD38 surface expression on NK subsets using primary TRPM2 antibody (1:10) at 2 h/1 h. (PDF 45 kb)

Additional file 13: Gating strategy for the analysis of TRPM2 and CD38 surface expression on NK subsets using primary TRPM2 antibody (1:5) at 2 h/1 h. (PDF 45 kb)

Additional file 14: Gating strategy for the analysis of TRPM2 and CD38 surface expression on unlabelled NK cells at 1 h/30 min. (PDF 39 kb)

Additional file 15: Gating strategy for the analysis of TRPM2 and CD38 surface expression on NK subsets using a secondary antibody control at 1 h/30 min. (PDF 40 kb)

Additional file 16: Gating strategy for the analysis of TRPM2 and CD38 surface expression on NK subsets using a fluorescence minus one control at 1 h/30 min. (PDF 42 kb)

Additional file 17: Gating strategy for the analysis of TRPM2 and CD38 surface expression on NK subsets using normal rabbit serum (1:300) at 1 h/30 min. (PDF 43 kb)

Additional file 18: Gating strategy for the analysis of TRPM2 and CD38 surface expression on NK subsets using normal rabbit serum (1:100) at 1 h/30 min. (PDF 44 kb)

Additional file 19: Gating strategy for the analysis of TRPM2 and CD38 surface expression on NK subsets using normal rabbit serum (1:50) at 1 h/30 min. (PDF 43 kb)

Additional file 20: Gating strategy for the analysis of TRPM2 and CD38 surface expression on NK subsets using normal rabbit serum (1:10) at 1 h/30 min. (PDF 44 kb)

Additional file 21: Gating strategy for the analysis of TRPM2 and CD38 surface expression on NK subsets using normal rabbit serum (1:5) at 1 h/30 min. (PDF 45 kb)

Additional file 22: Gating strategy for the analysis of TRPM2 and CD38 surface expression on NK subsets using primary TRPM2 antibody (1:300) at 1 h/30 min. (PDF 44 kb)

Additional file 23: Gating strategy for the analysis of TRPM2 and CD38 surface expression on NK subsets using primary TRPM2 antibody (1:100) at 1 h/30 min. (PDF 44 kb)

Additional file 24: Gating strategy for the analysis of TRPM2 and CD38 surface expression on NK subsets using primary TRPM2 antibody (1:50) at 1 h/30 min. (PDF 44 kb)

Additional file 25: Gating strategy for the analysis of TRPM2 and CD38 surface expression on NK subsets using primary TRPM2 antibody (1:10) at 1 h/30 min. (PDF 44 kb)

Additional file 26: Gating strategy for the analysis of TRPM2 and CD38 surface expression on NK subsets using primary TRPM2 antibody (1:5) at 1 h/30 min. (PDF 45 kb)

## Abbreviations

1°: primary
ADPR: Adenosine diphosphate-ribose
AMP: Adenosine monophosphate
BD: Becton Dickinson
Ca^2+^: Calcium
cADPR: Cyclic adenosine disphosphate-ribose
EDTA: Ethylenediaminetetraacetic acid
FcR: Fc receptor
FITC: Fluorescein isothiocyanate
FMO: Fluorescence minus one
NAADP: Nicotinic acid adenine dinucleotide phosphate
NCNED: National Centre of Neuroimmunology and Emerging Diseases
NK: Natural killer
PBMCs: Peripheral blood mononuclear cells
SEM: Standard error of the mean
TRPM2: Transient Receptor Potential Melastatin 2
