# Supplementary material for: Identification and characterisation of transient receptor potential melastatin 2 and CD38 channels on natural killer cells using the novel application of flow cytometry
Source: BMC Immunol. 2019 May 10;20:14. doi: 10.1186/s12865-019-0293-0 (PMC6509826; doi:10.1186/s12865-019-0293-0)
Supplement: Supplementary file 1 — Gating strategy for the analysis of TRPM2 and CD38 surface expression on unlabelled NK cells at 2 h/1 h. (PDF 39 kb) [file 12865_2019_293_MOESM1_ESM.pdf]

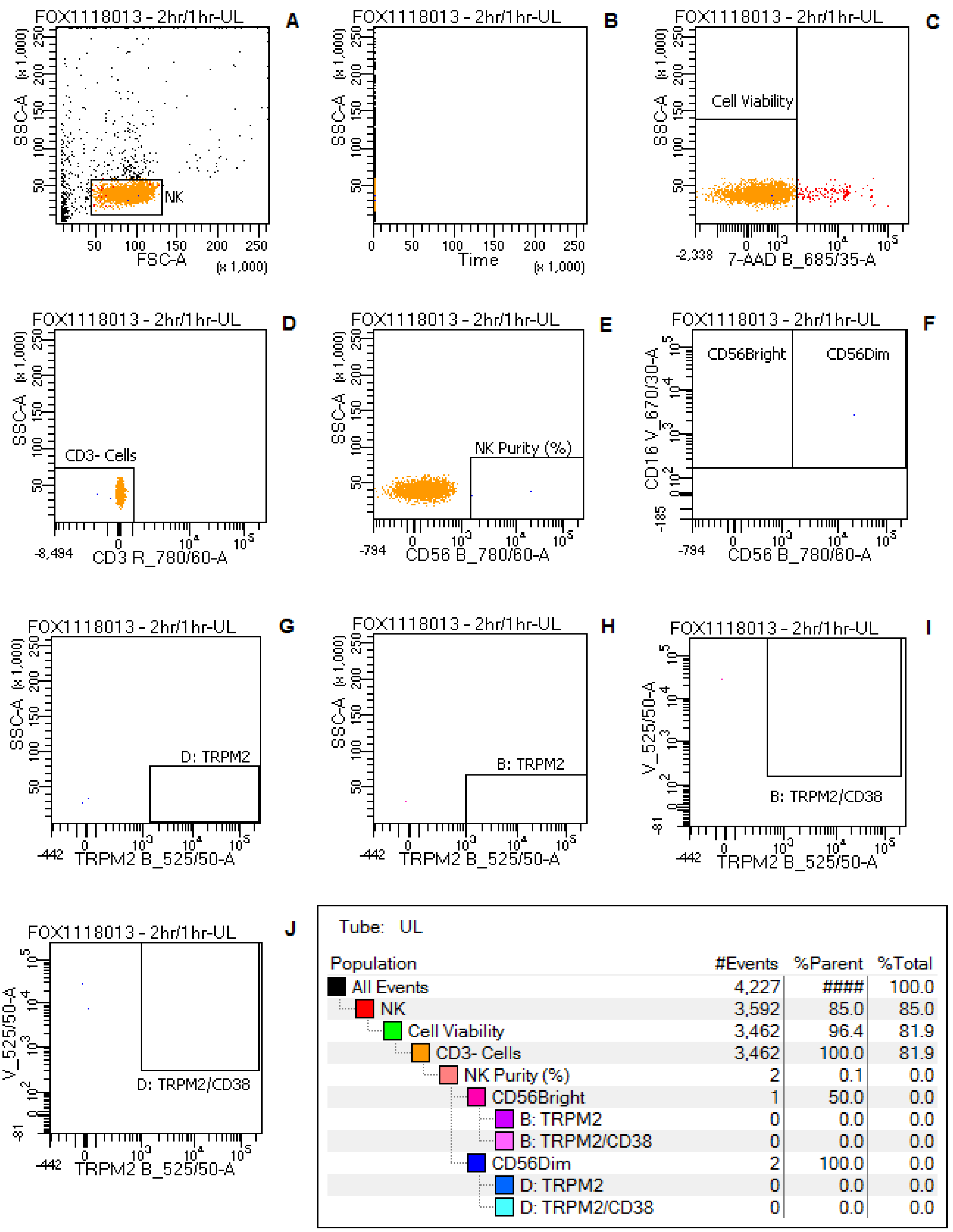

Population	#Events	%Parent	%Total
All Events	4,227	####	100.0
NK	3,592	85.0	85.0
Cell Viability	3,462	96.4	81.9
CD3- Cells	3,462	100.0	81.9
NK Purity (%)	2	0.1	0.0
CD56Bright	1	50.0	0.0
B: TRPM2	0	0.0	0.0
B: TRPM2/CD38	0	0.0	0.0
CD56Dim	2	100.0	0.0
D: TRPM2	0	0.0	0.0
D: TRPM2/CD38	0	0.0	0.0

Figure S1. Gating strategy of TRPM2 and CD38 using unlabelled natural killer cells at 2hr/1hr.

Additional file 1: **Figure S1.** Gating strategy of TRPM2 and CD38 using unlabelled natural killer cells at 2hr/1hr. **(a)** Lymphocyte populations were identified using forward and side scatter dot plots. (**b)** Time of acquirement was measured followed by **(c)** staining of 7-AAD to measure cell viability. **(d)** $CD3^{+}$ cells were excluded and only **(e)** $CD3^{-}$ lymphocytes were further used to characterise NK cells by CD56. **(f)** $CD3^{-}/CD56^{+}$ NK cells were sorted into $CD56^{Bright}CD16^{Dim/-}$ and $CD56^{Dim}CD16^{/+}$ NK cell subsets using CD56 and CD16. TRPM2 surface expression was measured on **(g)** $CD56^{Dim}CD16^{/+}$ and **(h)** $CD56^{Bright}CD16^{Dim/-}$ NK cell subsets. Dual surface expression of TRPM2 and CD38 was furthermore assessed on **(i)** $CD56^{Bright}CD16^{Dim/-}$ and **(j)** $CD56^{Dim}CD16^{/+}$ NK cell subsets. Data are represented as mean ± SEM. (PDF 40kb).
