# Supplementary material for: Identification and characterisation of transient receptor potential melastatin 2 and CD38 channels on natural killer cells using the novel application of flow cytometry
Source: BMC Immunol. 2019 May 10;20:14. doi: 10.1186/s12865-019-0293-0 (PMC6509826; doi:10.1186/s12865-019-0293-0)
Supplement: Supplementary file 5 — Gating strategy for the analysis of TRPM2 and CD38 surface expression on NK subsets using normal rabbit serum (1:100) at 2 h/1 h. (PDF 44 kb) [file 12865_2019_293_MOESM5_ESM.pdf]

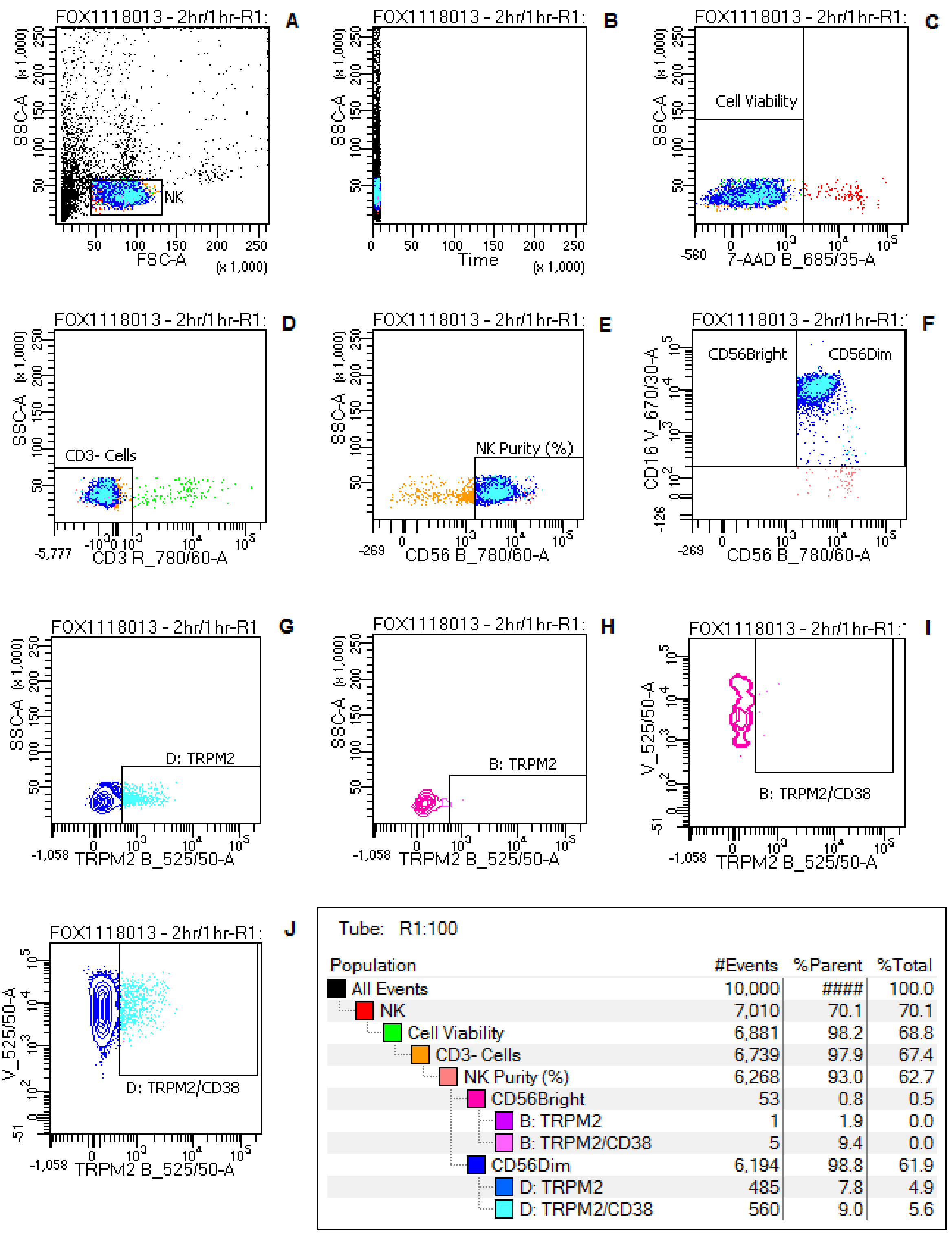

Population	#Events	%Parent	%Total
All Events	10,000	####	100.0
NK	7,010	70.1	70.1
Cell Viability	6,881	98.2	68.8
CD3- Cells	6,739	97.9	67.4
NK Purity (%)	6,268	93.0	62.7
CD56Bright	53	0.8	0.5
B: TRPM2	1	1.9	0.0
B: TRPM2/CD38	5	9.4	0.0
CD56Dim	6,194	98.8	61.9
D: TRPM2	485	7.8	4.9
D: TRPM2/CD38	560	9.0	5.6

Figure S5. Gating strategy of TRPM2 and CD38 using normal rabbit serum (1:100) at 2hr/1hr.

Additional file 5: **Figure S5.** Gating strategy of TRPM2 and CD38 using normal rabbit serum (1:100) at 2hr/1hr. **(a)** Lymphocyte populations were identified using forward and side scatter dot plots. (**b)** Duration of cell acquirement was measured followed by **(c)** cell viability after 7-AAD staining. **(d)** $CD3^+$ cells were excluded and only **(e)** $CD3^-$ lymphocytes were further used to characterise NK cells by CD56. **(f)** $CD3^-/CD56^+$ NK cells were sorted into $CD56^{Bright}CD16^{Dim/-}$ and $CD56^{Dim}CD16^{/+}$ NK cell subsets using CD56 and CD16. TRPM2 surface expression was measured on **(g)** $CD56^{Dim}CD16^{/+}$ and **(h)** $CD56^{Bright}CD16^{Dim/-}$ NK cell subsets. Dual surface expression of TRPM2 and CD38 was furthermore assessed on **(i)** $CD56^{Bright}CD16^{Dim/-}$ and **(j)** $CD56^{Dim}CD16^{/+}$ NK cell subsets. Data are represented as mean ± SEM. (PDF 45kb).
