# Supplementary material for: Identification and characterisation of transient receptor potential melastatin 2 and CD38 channels on natural killer cells using the novel application of flow cytometry
Source: BMC Immunol. 2019 May 10;20:14. doi: 10.1186/s12865-019-0293-0 (PMC6509826; doi:10.1186/s12865-019-0293-0)
Supplement: Supplementary file 9 — Gating strategy for the analysis of TRPM2 and CD38 surface expression on NK subsets using primary TRPM2 antibody (1:300) at 2 h/1 h. (PDF 43 kb) [file 12865_2019_293_MOESM9_ESM.pdf]

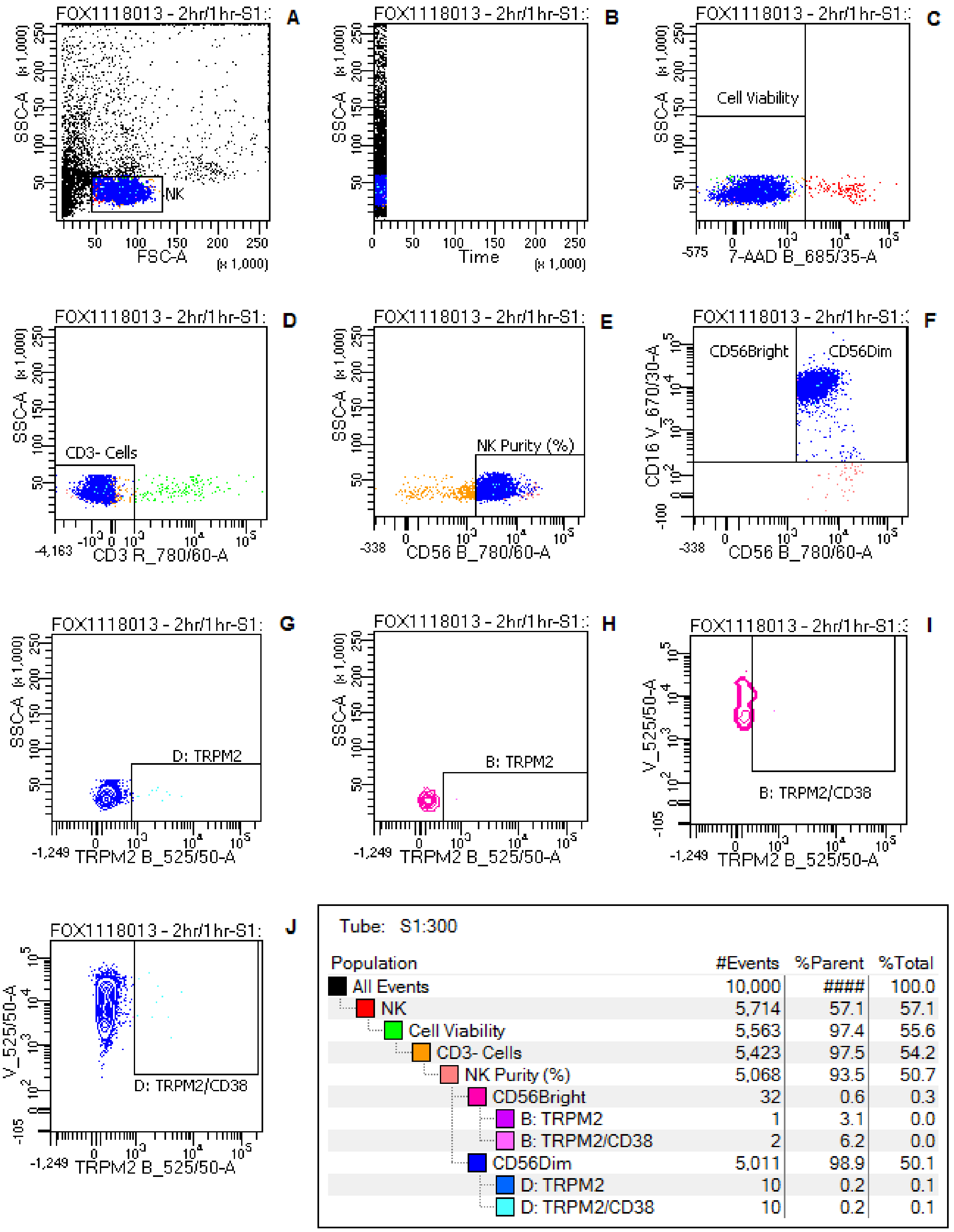

Tube: S1:300

Population	#Events	%Parent	%Total
All Events	10,000	####	100.0
NK	5,714	57.1	57.1
Cell Viability	5,563	97.4	55.6
CD3- Cells	5,423	97.5	54.2
NK Purity (%)	5,068	93.5	50.7
CD56Bright	32	0.6	0.3
B: TRPM2	1	3.1	0.0
B: TRPM2/CD38	2	6.2	0.0
CD56Dim	5,011	98.9	50.1
D: TRPM2	10	0.2	0.1
D: TRPM2/CD38	10	0.2	0.1

Figure S9. Gating strategy of TRPM2 and CD38 using primary TRPM2 antibody (1:300) at 2hr/1hr.

Additional file 9: **Figure S9.** Gating strategy of TRPM2 and CD38 using primary TRPM2 antibody (1:300) at 2hr/1hr. **(a)** Lymphocyte populations were identified using forward and side scatter dot plots. (**b)** Duration of cell acquirement was measured followed by **(c)** cell viability after 7-AAD staining. **(d)** $CD3^{+}$ cells were excluded and only **(e)** $CD3^{-}$ lymphocytes were further used to characterise NK cells by CD56. **(f)** $CD3^{-}/CD56^{+}$ NK cells were sorted into $CD56^{Bright}CD16^{Dim/-}$ and $CD56^{Dim}CD16^{/+}$ NK cell subsets using CD56 and CD16. TRPM2 surface expression was measured on **(g)** $CD56^{Dim}CD16^{/+}$ and **(h)** $CD56^{Bright}CD16^{Dim/-}$ NK cell subsets. Dual surface expression of TRPM2 and CD38 was furthermore assessed on **(i)** $CD56^{Bright}CD16^{Dim/-}$ and **(j)** $CD56^{Dim}CD16^{/+}$ NK cell subsets. Data are represented as mean ± SEM. (PDF 44kb).
