# Supplementary material for: Identification and characterisation of transient receptor potential melastatin 2 and CD38 channels on natural killer cells using the novel application of flow cytometry
Source: BMC Immunol. 2019 May 10;20:14. doi: 10.1186/s12865-019-0293-0 (PMC6509826; doi:10.1186/s12865-019-0293-0)
Supplement: Supplementary file 10 — Gating strategy for the analysis of TRPM2 and CD38 surface expression on NK subsets using primary TRPM2 antibody (1:100) at 2 h/1 h. (PDF 44 kb) [file 12865_2019_293_MOESM10_ESM.pdf]

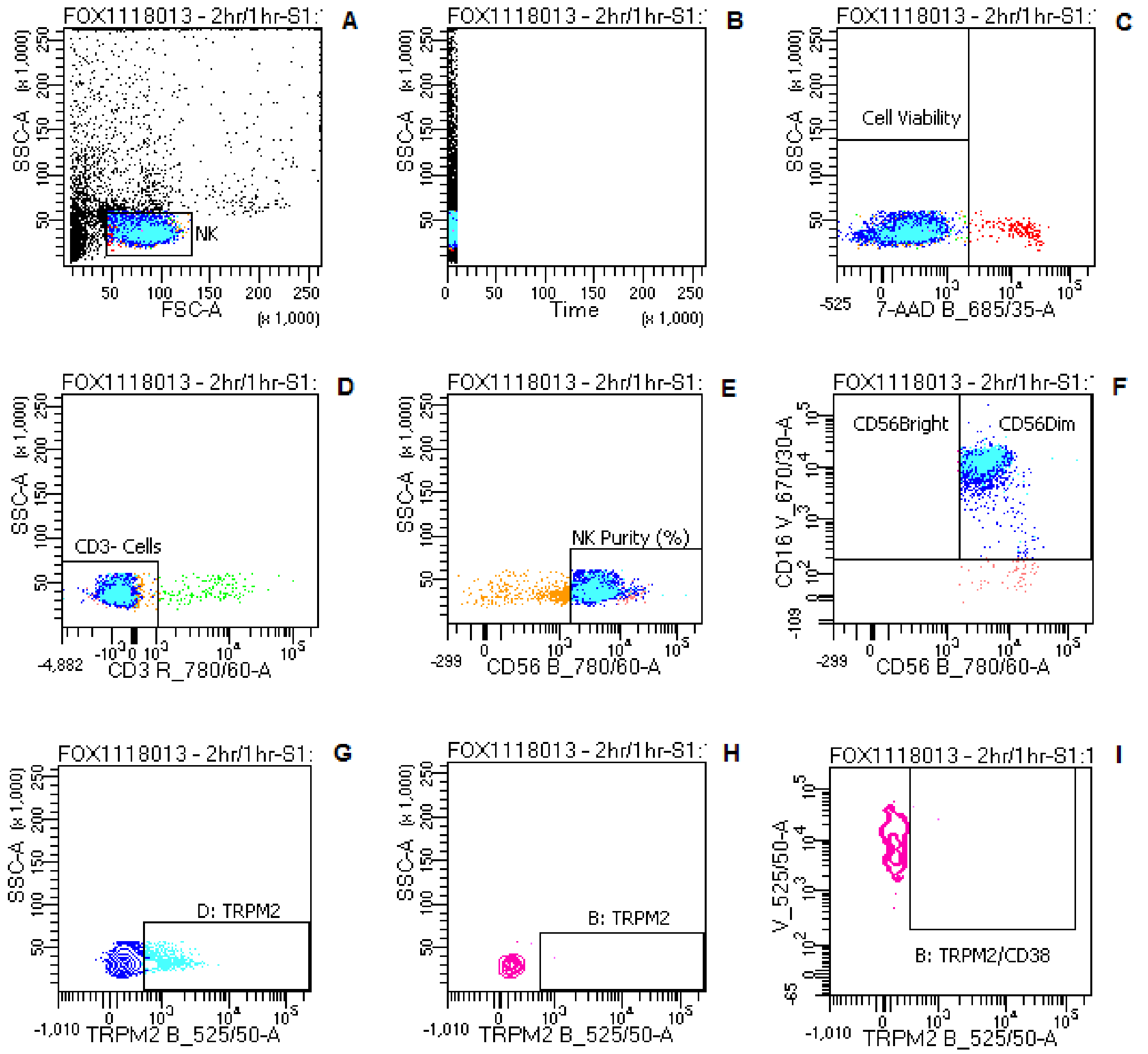

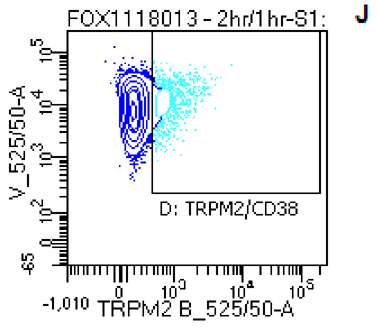

Tube: S1:100

Population	#Events	%Parent	%Total
All Events	10,000	####	100.0
NK	6,883	68.8	68.8
Cell Viability	6,693	97.2	66.9
CD3- Cells	6,545	97.8	65.4
NK Purity (%)	6,066	92.7	60.7
CD56Bright	46	0.8	0.5
B: TRPM2	1	2.2	0.0
B: TRPM2/CD38	2	4.3	0.0
CD56Dim	5,986	98.7	59.9
D: TRPM2	776	13.0	7.8
D: TRPM2/CD38	886	14.8	8.9

Figure S10. Gating strategy of TRPM2 and CD38 using primary TRPM2 antibody (1:100) at 2hr/1hr.

Additional file 10: **Figure S10.** Gating strategy of TRPM2 and CD38 using primary TRPM2 antibody (1:100) at 2hr/1hr. **(a)** Lymphocyte populations were identified using forward and side scatter dot plots. (**b)** Duration of cell acquirement was measured followed by **(c)** cell viability after 7-AAD staining. **(d)** $CD3^{+}$ cells were excluded and only **(e)** $CD3^{-}$ lymphocytes were further used to characterise NK cells by CD56. **(f)** $CD3^{-}/CD56^{+}$ NK cells were sorted into $CD56^{Bright}CD16^{Dim/-}$ and $CD56^{Dim}CD16^{/+}$ NK cell subsets using CD56 and CD16. TRPM2 surface expression was measured on **(g)** $CD56^{Dim}CD16^{/+}$ and **(h)** $CD56^{Bright}CD16^{Dim/-}$ NK cell subsets. Dual surface expression of TRPM2 and CD38 was furthermore assessed on **(i)** $CD56^{Bright}CD16^{Dim/-}$ and **(j)** $CD56^{Dim}CD16^{/+}$ NK cell subsets. Data are represented as mean ± SEM. (PDF 45kb).
