# Supplementary material for: Identification and characterisation of transient receptor potential melastatin 2 and CD38 channels on natural killer cells using the novel application of flow cytometry
Source: BMC Immunol. 2019 May 10;20:14. doi: 10.1186/s12865-019-0293-0 (PMC6509826; doi:10.1186/s12865-019-0293-0)
Supplement: Supplementary file 11 — Gating strategy for the analysis of TRPM2 and CD38 surface expression on NK subsets using primary TRPM2 antibody (1:50) at 2 h/1 h. (PDF 44 kb) [file 12865_2019_293_MOESM11_ESM.pdf]

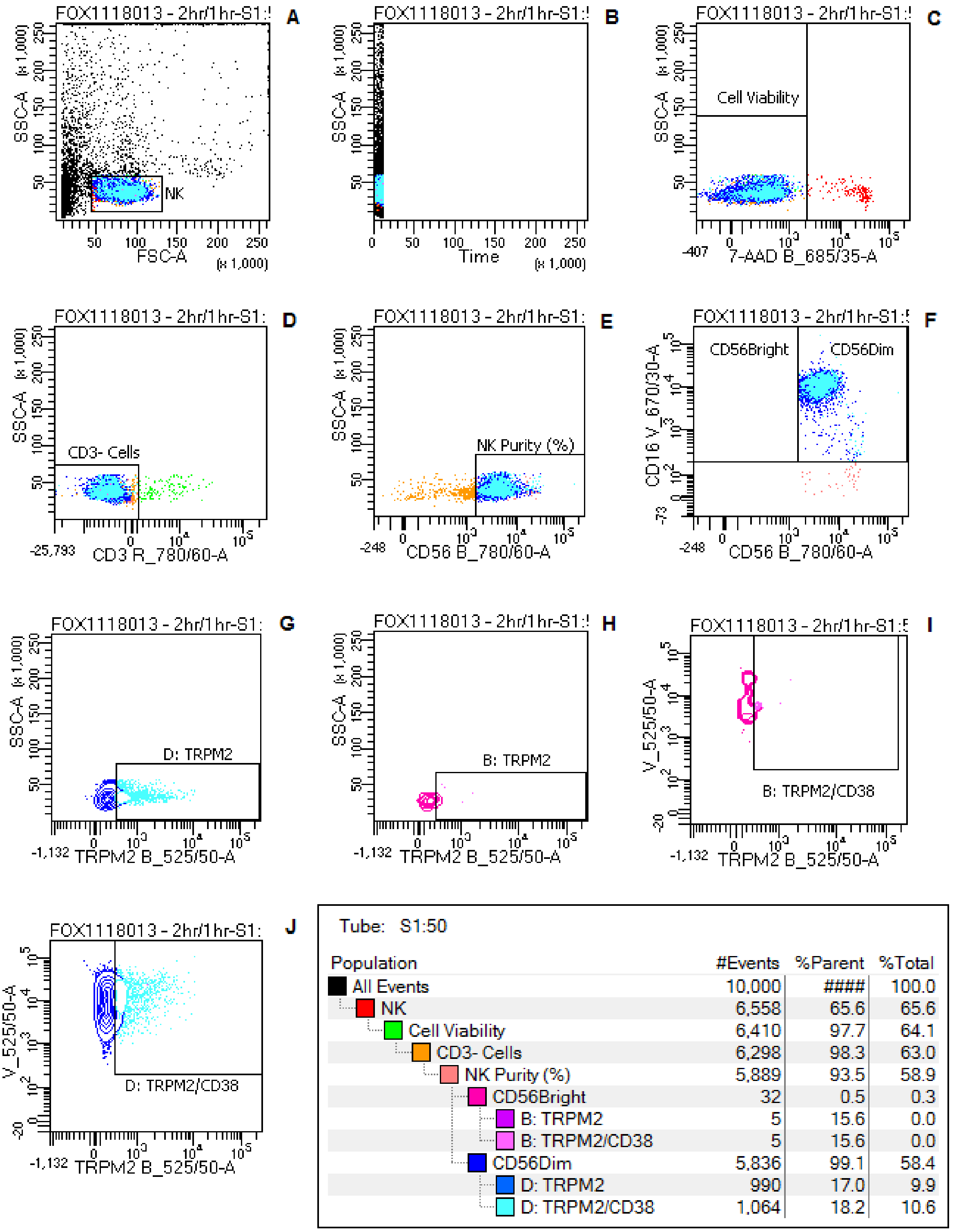

Tube: S1:50

Population	#Events	%Parent	%Total
All Events	10,000	####	100.0
NK	6,558	65.6	65.6
Cell Viability	6,410	97.7	64.1
CD3- Cells	6,298	98.3	63.0
NK Purity (%)	5,889	93.5	58.9
CD56Bright	32	0.5	0.3
B: TRPM2	5	15.6	0.0
B: TRPM2/CD38	5	15.6	0.0
CD56Dim	5,836	99.1	58.4
D: TRPM2	990	17.0	9.9
D: TRPM2/CD38	1,064	18.2	10.6

Figure S11. Gating strategy of TRPM2 and CD38 using primary TRPM2 antibody (1:50) at 2hr/1hr.

Additional file 11: **Figure S11.** Gating strategy of TRPM2 and CD38 using primary TRPM2 antibody (1:50) at 2hr/1hr. **(a)** Lymphocyte populations were identified using forward and side scatter dot plots. (**b)** Duration of cell acquirement was measured followed by **(c)** cell viability after 7-AAD staining. **(d)** $CD3^{+}$ cells were excluded and only **(e)** $CD3^{-}$ lymphocytes were further used to characterise NK cells by CD56. **(f)** $CD3^{-}/CD56^{+}$ NK cells were sorted into $CD56^{Bright}CD16^{Dim/-}$ and $CD56^{Dim}CD16^{/+}$ NK cell subsets using CD56 and CD16. TRPM2 surface expression was measured on **(g)** $CD56^{Dim}CD16^{/+}$ and **(h)** $CD56^{Bright}CD16^{Dim/-}$ NK cell subsets. Dual surface expression of TRPM2 and CD38 was furthermore assessed on **(i)** $CD56^{Bright}CD16^{Dim/-}$ and **(j)** $CD56^{Dim}CD16^{/+}$ NK cell subsets. Data are represented as mean ± SEM. (PDF 45kb).
