# Supplementary material for: Identification and characterisation of transient receptor potential melastatin 2 and CD38 channels on natural killer cells using the novel application of flow cytometry
Source: BMC Immunol. 2019 May 10;20:14. doi: 10.1186/s12865-019-0293-0 (PMC6509826; doi:10.1186/s12865-019-0293-0)
Supplement: Supplementary file 13 — Gating strategy for the analysis of TRPM2 and CD38 surface expression on NK subsets using primary TRPM2 antibody (1:5) at 2 h/1 h. (PDF 45 kb) [file 12865_2019_293_MOESM13_ESM.pdf]

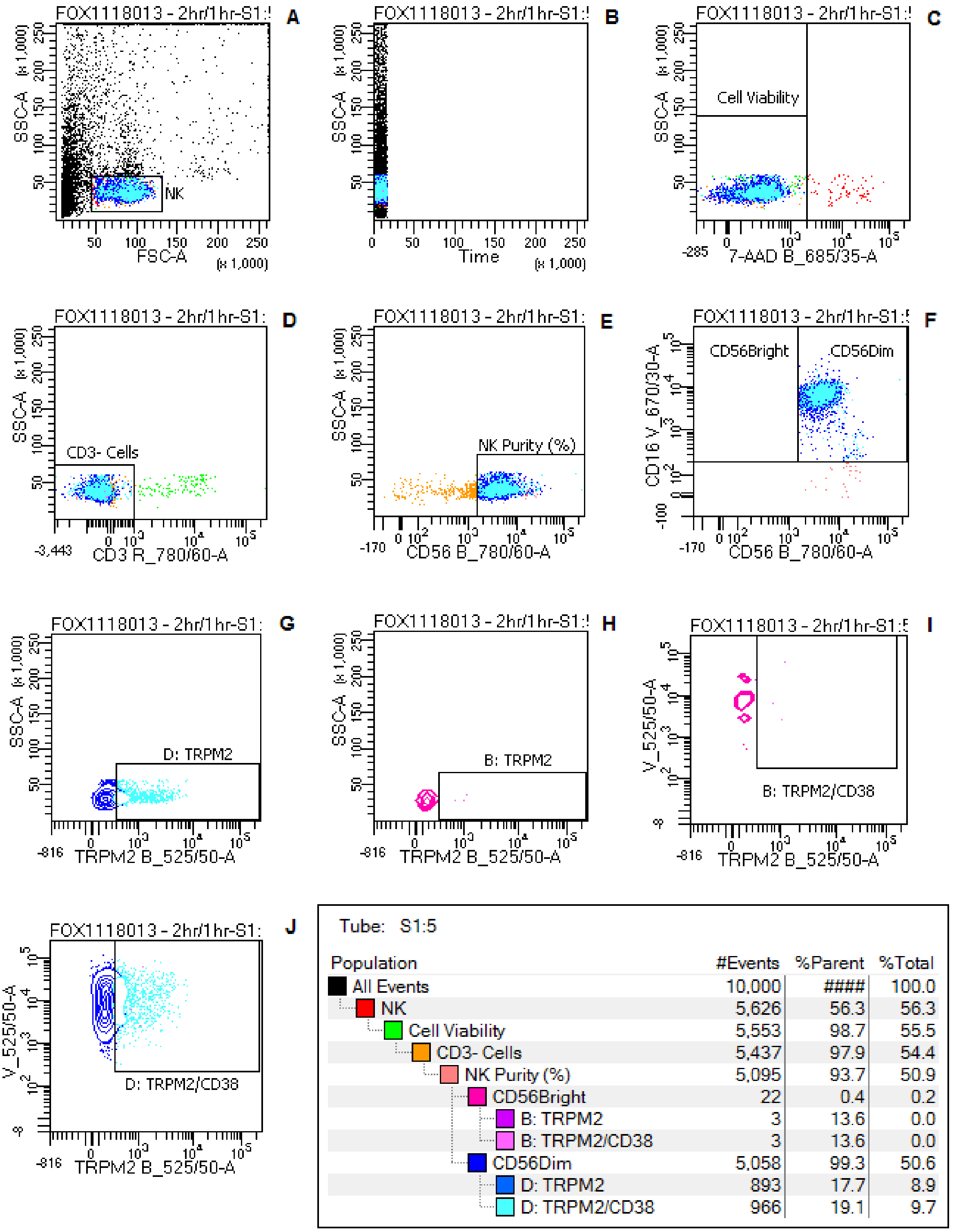

Population	#Events	%Parent	%Total
All Events	10,000	####	100.0
NK	5,626	56.3	56.3
Cell Viability	5,553	98.7	55.5
CD3- Cells	5,437	97.9	54.4
NK Purity (%)	5,095	93.7	50.9
CD56Bright	22	0.4	0.2
B: TRPM2	3	13.6	0.0
B: TRPM2/CD38	3	13.6	0.0
CD56Dim	5,058	99.3	50.6
D: TRPM2	893	17.7	8.9
D: TRPM2/CD38	966	19.1	9.7

Figure S13. Gating strategy of TRPM2 and CD38 using primary TRPM2 antibody (1:5) at 2hr/1hr.

Additional file 13: **Figure S13.** Gating strategy of TRPM2 and CD38 using primary TRPM2 antibody (1:5) at 2hr/1hr. **(a)** Lymphocyte populations were identified using forward and side scatter dot plots. (**b)** Duration of cell acquirement was measured followed by **(c)** cell viability after 7-AAD staining. **(d)** $CD3^{+}$ cells were excluded and only **(e)** $CD3^{-}$ lymphocytes were further used to characterise NK cells by CD56. **(f)** $CD3^{-}/CD56^{+}$ NK cells were sorted into $CD56^{Bright}CD16^{Dim/-}$ and $CD56^{Dim}CD16^{/+}$ NK cell subsets using CD56 and CD16. TRPM2 surface expression was measured on **(g)** $CD56^{Dim}CD16^{/+}$ and **(h)** $CD56^{Bright}CD16^{Dim/-}$ NK cell subsets. Dual surface expression of TRPM2 and CD38 was furthermore assessed on **(i)** $CD56^{Bright}CD16^{Dim/-}$ and **(j)** $CD56^{Dim}CD16^{/+}$ NK cell subsets. Data are represented as mean ± SEM. (PDF 46kb).
