# Supplementary material for: Identification and characterisation of transient receptor potential melastatin 2 and CD38 channels on natural killer cells using the novel application of flow cytometry
Source: BMC Immunol. 2019 May 10;20:14. doi: 10.1186/s12865-019-0293-0 (PMC6509826; doi:10.1186/s12865-019-0293-0)
Supplement: Supplementary file 15 — Gating strategy for the analysis of TRPM2 and CD38 surface expression on NK subsets using a secondary antibody control at 1 h/30 min. (PDF 40 kb) [file 12865_2019_293_MOESM15_ESM.pdf]

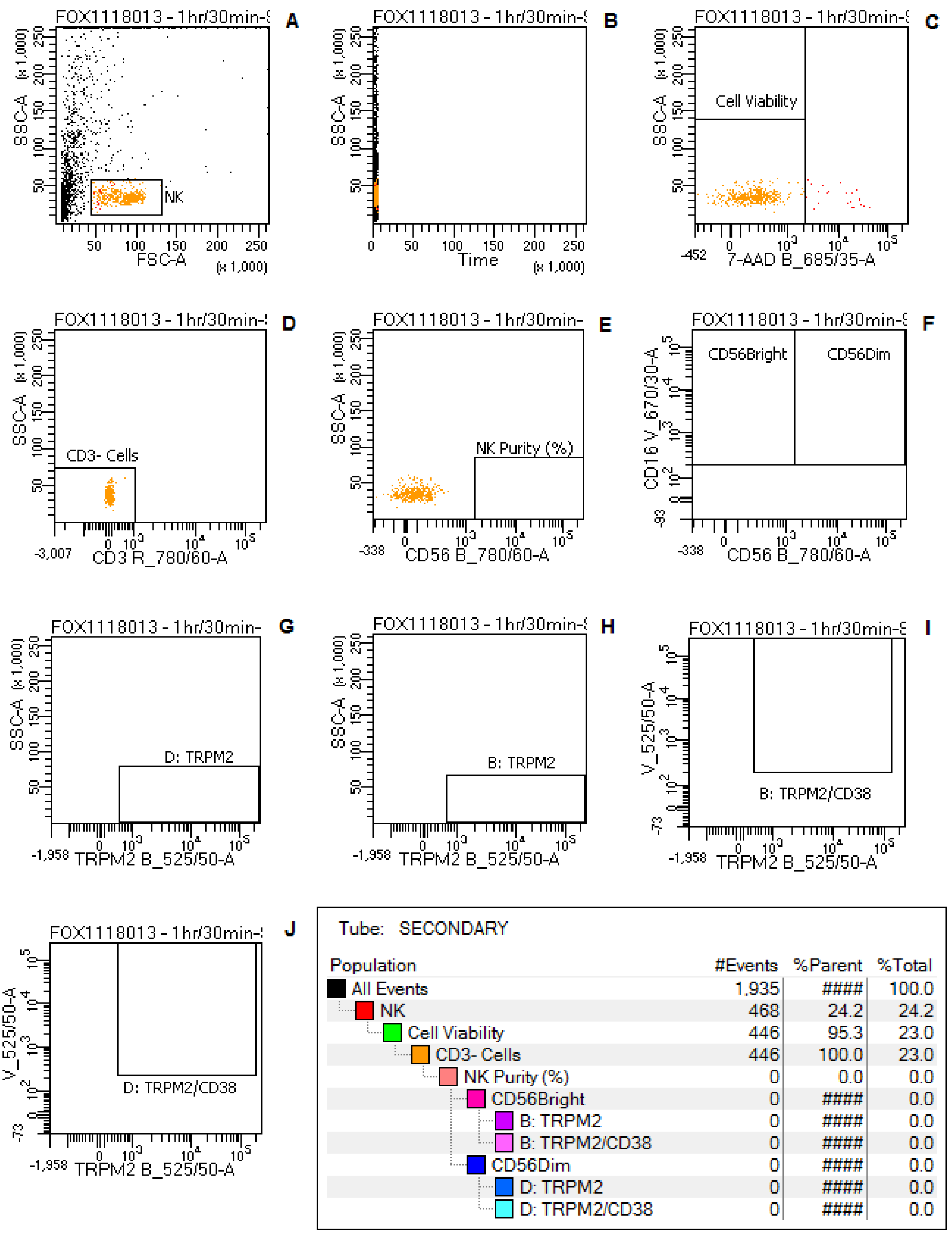

Tube: SECONDARY

Population	#Events	%Parent	%Total
All Events	1,935	####	100.0
NK	468	24.2	24.2
Cell Viability	446	95.3	23.0
CD3- Cells	446	100.0	23.0
NK Purity (%)	0	0.0	0.0
CD56Bright	0	####	0.0
B: TRPM2	0	####	0.0
B: TRPM2/CD38	0	####	0.0
CD56Dim	0	####	0.0
D: TRPM2	0	####	0.0
D: TRPM2/CD38	0	####	0.0

Figure S15. Gating strategy of TRPM2 and CD38 using secondary antibody only at 1hr/30min.

Additional file 15: **Figure S15.** Gating strategy of TRPM2 and CD38 using secondary antibody only at 1hr/30min. **(a)** Lymphocyte populations were identified using forward and side scatter dot plots. (**b)** Duration of cell acquirement was measured followed by **(c)** cell viability after 7-AAD staining. **(d)** $CD3^{+}$ cells were excluded and only **(e)** $CD3^{-}$ lymphocytes were further used to characterise NK cells by CD56. **(f)** $CD3^{-}/CD56^{+}$ NK cells were sorted into $CD56^{Bright}CD16^{Dim/-}$ and $CD56^{Dim}CD16^{/+}$ NK cell subsets using CD56 and CD16. TRPM2 surface expression was measured on **(g)** $CD56^{Dim}CD16^{/+}$ and **(h)** $CD56^{Bright}CD16^{Dim/-}$ NK cell subsets. Dual surface expression of TRPM2 and CD38 was furthermore assessed on **(i)** $CD56^{Bright}CD16^{Dim/-}$ and **(j)** $CD56^{Dim}CD16^{/+}$ NK cell subsets. Data are represented as mean ± SEM. (PDF 41kb).
