# Supplementary material for: Identification and characterisation of transient receptor potential melastatin 2 and CD38 channels on natural killer cells using the novel application of flow cytometry
Source: BMC Immunol. 2019 May 10;20:14. doi: 10.1186/s12865-019-0293-0 (PMC6509826; doi:10.1186/s12865-019-0293-0)
Supplement: Supplementary file 17 — Gating strategy for the analysis of TRPM2 and CD38 surface expression on NK subsets using normal rabbit serum (1:300) at 1 h/30 min. (PDF 43 kb) [file 12865_2019_293_MOESM17_ESM.pdf]

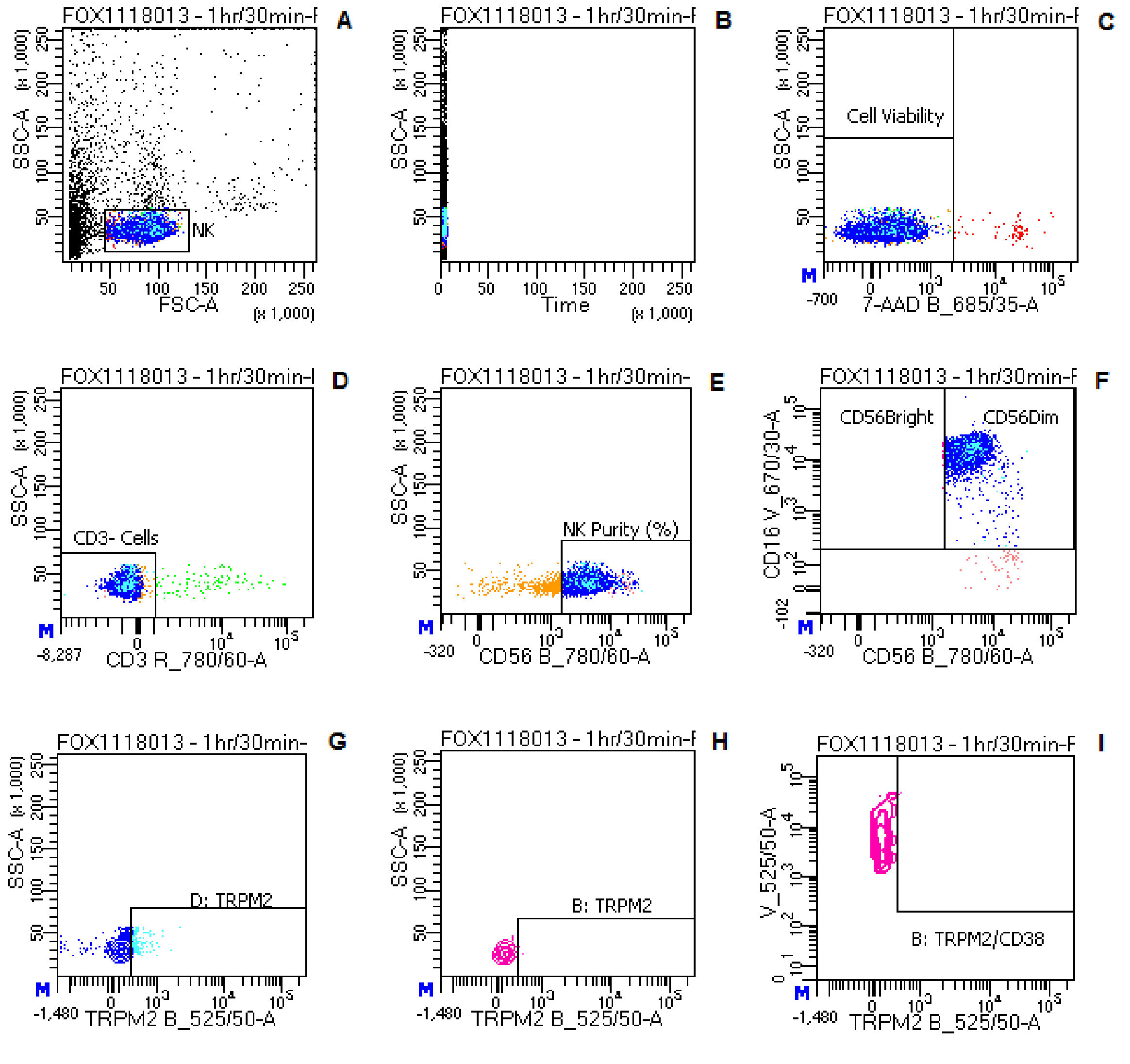

Tube: R1:300

Population	#Events	%Parent	%Total
All Events	10,000	####	100.0
NK	7,258	72.6	72.6
Cell Viability	7,188	99.0	71.9
CD3- Cells	7,083	98.5	70.8
NK Purity (%)	6,561	92.6	65.6
CD56Bright	70	1.1	0.7
B: TRPM2	1	1.4	0.0
B: TRPM2/CD38	1	1.4	0.0
CD56Dim	6,485	98.8	64.8
D: TRPM2	116	1.8	1.2
D: TRPM2/CD38	134	2.1	1.3

Figure S17. Gating strategy of TRPM2 and CD38 using normal rabbit serum (1:300) at 1hr/30min.

Additional file 17: **Figure S17.** Gating strategy of TRPM2 and CD38 using normal rabbit serum (1:300) at 1hr/30min. **(a)** Lymphocyte populations were identified using forward and side scatter dot plots. (**b)** Duration of cell acquirement was measured followed by **(c)** cell viability after 7-AAD staining. **(d)** $CD3^{+}$ cells were excluded and only **(e)** $CD3^{-}$ lymphocytes were further used to characterise NK cells by CD56. **(f)** $CD3^{-}/CD56^{+}$ NK cells were sorted into $CD56^{Bright}CD16^{Dim/-}$ and $CD56^{Dim}CD16^{/+}$ NK cell subsets using CD56 and CD16. TRPM2 surface expression was measured on **(g)** $CD56^{Dim}CD16^{/+}$ and **(h)** $CD56^{Bright}CD16^{Dim/-}$ NK cell subsets. Dual surface expression of TRPM2 and CD38 was furthermore assessed on **(i)** $CD56^{Bright}CD16^{Dim/-}$ and **(j)** $CD56^{Dim}CD16^{/+}$ NK cell subsets. Data are represented as mean ± SEM. (PDF 44kb).
