# Supplementary material for: Identification and characterisation of transient receptor potential melastatin 2 and CD38 channels on natural killer cells using the novel application of flow cytometry
Source: BMC Immunol. 2019 May 10;20:14. doi: 10.1186/s12865-019-0293-0 (PMC6509826; doi:10.1186/s12865-019-0293-0)
Supplement: Supplementary file 20 — Gating strategy for the analysis of TRPM2 and CD38 surface expression on NK subsets using normal rabbit serum (1:10) at 1 h/30 min. (PDF 44 kb) [file 12865_2019_293_MOESM20_ESM.pdf]

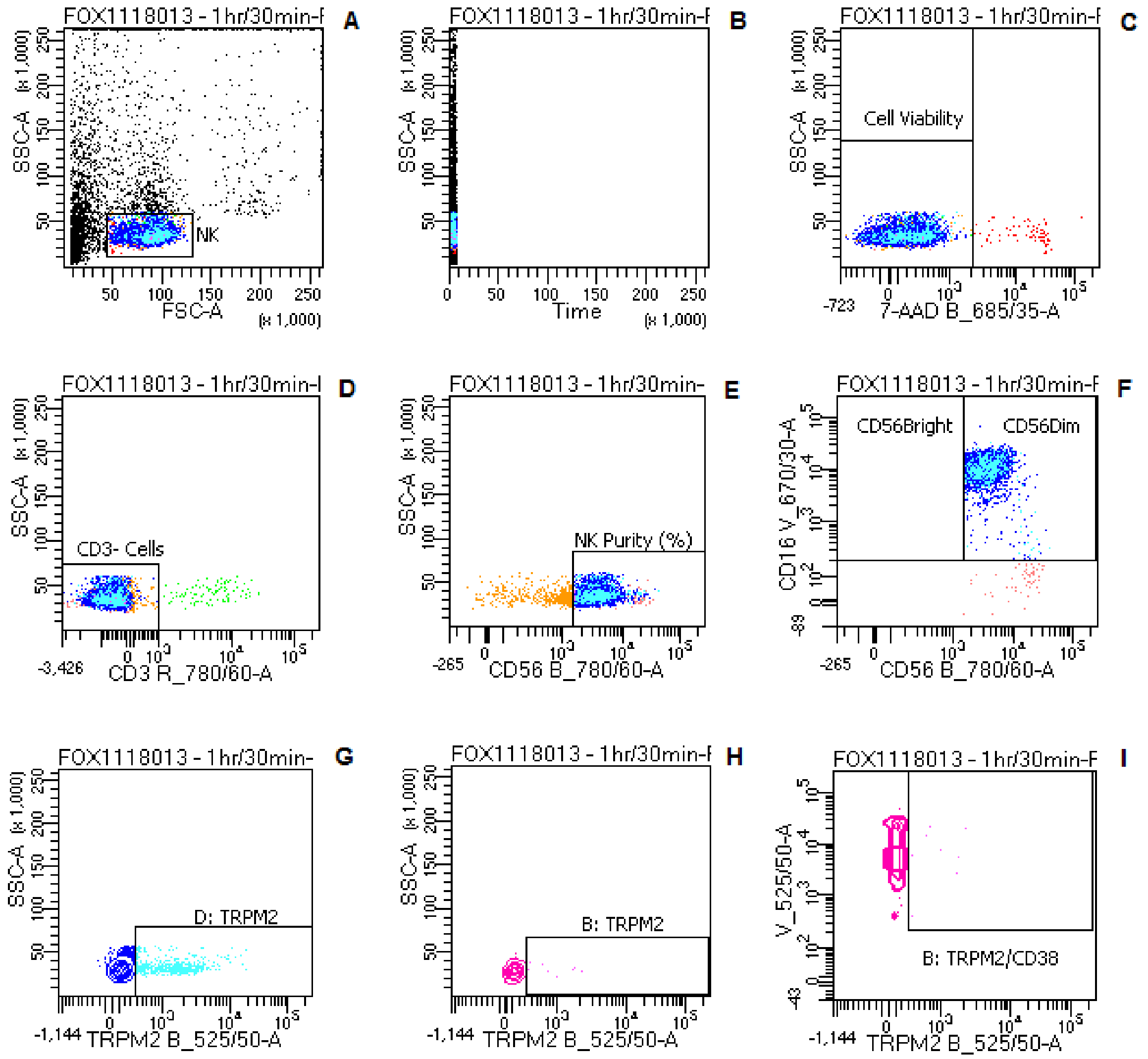

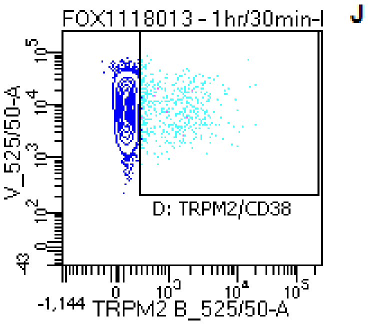

Tube: R1:10

Population	#Events	%Parent	%Total
All Events	10,000	####	100.0
NK	6,830	68.3	68.3
Cell Viability	6,748	98.8	67.5
CD3- Cells	6,653	98.6	66.5
NK Purity (%)	6,098	91.7	61.0
CD56Bright	70	1.1	0.7
B: TRPM2	7	10.0	0.1
B: TRPM2/CD38	8	11.4	0.1
CD56Dim	6,019	98.7	60.2
D: TRPM2	436	7.2	4.4
D: TRPM2/CD38	479	8.0	4.8

Figure S20. Gating strategy of TRPM2 and CD38 using normal rabbit serum (1:10) at 1hr/30min.

Additional file 20: **Figure S20.** Gating strategy of TRPM2 and CD38 using normal rabbit serum (1:10) at 1hr/30min. **(a)** Lymphocyte populations were identified using forward and side scatter dot plots. (**b)** Duration of cell acquirement was measured followed by **(c)** cell viability after 7-AAD staining. **(d)** $CD3^{+}$ cells were excluded and only **(e)** $CD3^{-}$ lymphocytes were further used to characterise NK cells by CD56. **(f)** $CD3^{-}/CD56^{+}$ NK cells were sorted into $CD56^{Bright}CD16^{Dim/-}$ and $CD56^{Dim}CD16^{/+}$ NK cell subsets using CD56 and CD16. TRPM2 surface expression was measured on **(g)** $CD56^{Dim}CD16^{/+}$ and **(h)** $CD56^{Bright}CD16^{Dim/-}$ NK cell subsets. Dual surface expression of TRPM2 and CD38 was furthermore assessed on **(i)** $CD56^{Bright}CD16^{Dim/-}$ and **(j)** $CD56^{Dim}CD16^{/+}$ NK cell subsets. Data are represented as mean ± SEM. (PDF 45kb).
