# Supplementary material for: Identification and characterisation of transient receptor potential melastatin 2 and CD38 channels on natural killer cells using the novel application of flow cytometry
Source: BMC Immunol. 2019 May 10;20:14. doi: 10.1186/s12865-019-0293-0 (PMC6509826; doi:10.1186/s12865-019-0293-0)
Supplement: Supplementary file 21 — Gating strategy for the analysis of TRPM2 and CD38 surface expression on NK subsets using normal rabbit serum (1:5) at 1 h/30 min. (PDF 45 kb) [file 12865_2019_293_MOESM21_ESM.pdf]

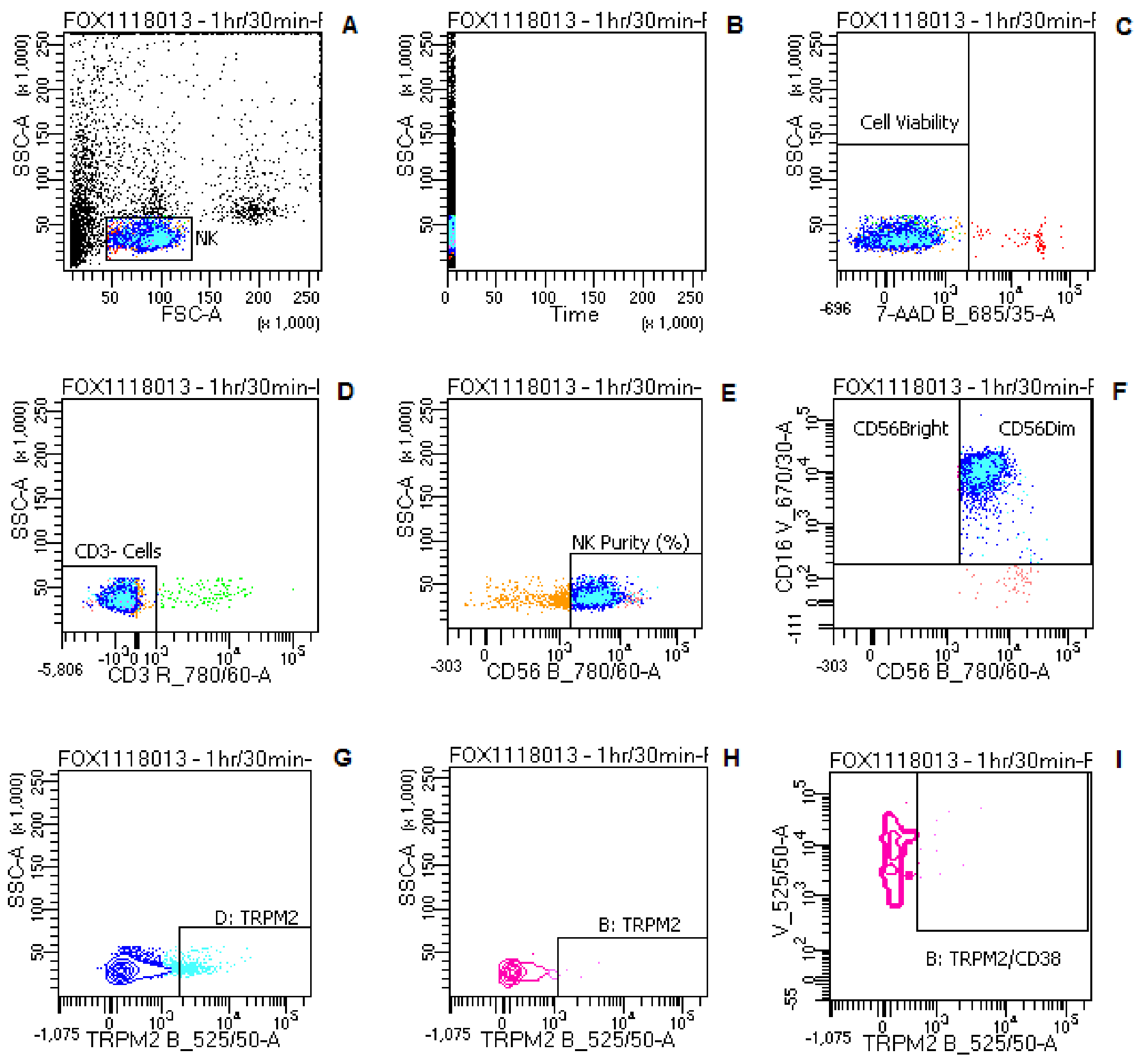

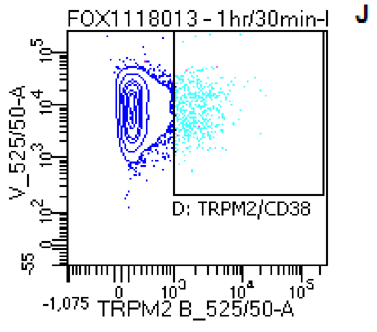

Tube: R1:5

Population	#Events	%Parent	%Total
All Events	10,000	####	100.0
NK	6,130	61.3	61.3
Cell Viability	6,046	98.6	60.5
CD3- Cells	5,930	98.1	59.3
NK Purity (%)	5,397	91.0	54.0
CD56Bright	67	1.2	0.7
B: TRPM2	3	4.5	0.0
B: TRPM2/CD38	9	13.4	0.1
CD56Dim	5,311	98.4	53.1
D: TRPM2	279	5.3	2.8
D: TRPM2/CD38	485	9.1	4.9

Figure S21. Gating strategy of TRPM2 and CD38 using normal rabbit serum (1:5) at 1hr/30min.

Additional file 21: **Figure S21.** Gating strategy of TRPM2 and CD38 using normal rabbit serum (1:5) at 1hr/30min. **(a)** Lymphocyte populations were identified using forward and side scatter dot plots. (**b)** Duration of cell acquirement was measured followed by **(c)** cell viability after 7-AAD staining. **(d)** $CD3^{+}$ cells were excluded and only **(e)** $CD3^{-}$ lymphocytes were further used to characterise NK cells by CD56. **(f)** $CD3^{-}/CD56^{+}$ NK cells were sorted into $CD56^{Bright}CD16^{Dim/-}$ and $CD56^{Dim}CD16^{/+}$ NK cell subsets using CD56 and CD16. TRPM2 surface expression was measured on **(g)** $CD56^{Dim}CD16^{/+}$ and **(h)** $CD56^{Bright}CD16^{Dim/-}$ NK cell subsets. Dual surface expression of TRPM2 and CD38 was furthermore assessed on **(i)** $CD56^{Bright}CD16^{Dim/-}$ and **(j)** $CD56^{Dim}CD16^{/+}$ NK cell subsets. Data are represented as mean ± SEM. (PDF 46kb).
