# Supplementary material for: Identification and characterisation of transient receptor potential melastatin 2 and CD38 channels on natural killer cells using the novel application of flow cytometry
Source: BMC Immunol. 2019 May 10;20:14. doi: 10.1186/s12865-019-0293-0 (PMC6509826; doi:10.1186/s12865-019-0293-0)
Supplement: Supplementary file 24 — Gating strategy for the analysis of TRPM2 and CD38 surface expression on NK subsets using primary TRPM2 antibody (1:50) at 1 h/30 min. (PDF 44 kb) [file 12865_2019_293_MOESM24_ESM.pdf]

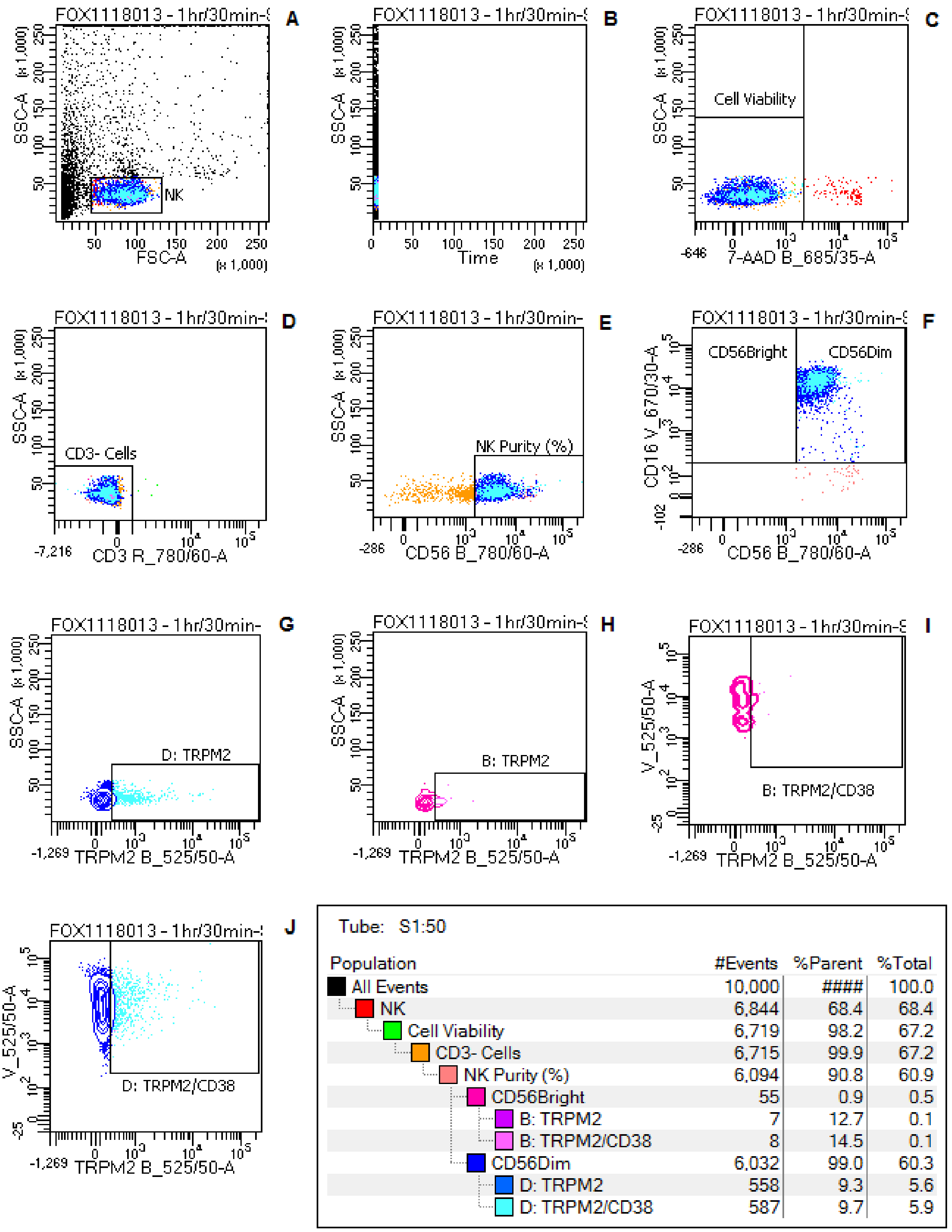

Tube: S1:50

Population	#Events	%Parent	%Total
All Events	10,000	####	100.0
NK	6,844	68.4	68.4
Cell Viability	6,719	98.2	67.2
CD3- Cells	6,715	99.9	67.2
NK Purity (%)	6,094	90.8	60.9
CD56Bright	55	0.9	0.5
B: TRPM2	7	12.7	0.1
B: TRPM2/CD38	8	14.5	0.1
CD56Dim	6,032	99.0	60.3
D: TRPM2	558	9.3	5.6
D: TRPM2/CD38	587	9.7	5.9

Figure S24. Gating strategy of TRPM2 and CD38 using primary TRPM2 antibody (1:50) at 1hr/30min.

Additional file 24: **Figure S24.** Gating strategy of TRPM2 and CD38 using primary TRPM2 antibody (1:50) at 1hr/30min. **(a)** Lymphocyte populations were identified using forward and side scatter dot plots. (**b)** Duration of cell acquirement was measured followed by **(c)** cell viability after 7-AAD staining. **(d)** $CD3^+$ cells were excluded and only **(e)** $CD3^-$ lymphocytes were further used to characterise NK cells by CD56. **(f)** $CD3^-/CD56^+$ NK cells were sorted into $CD56^{Bright}CD16^{Dim/-}$ and $CD56^{Dim}CD16^{/+}$ NK cell subsets using CD56 and CD16. TRPM2 surface expression was measured on **(g)** $CD56^{Dim}CD16^{/+}$ and **(h)** $CD56^{Bright}CD16^{Dim/-}$ NK cell subsets. Dual surface expression of TRPM2 and CD38 was furthermore assessed on **(i)** $CD56^{Bright}CD16^{Dim/-}$ and **(j)** $CD56^{Dim}CD16^{/+}$ NK cell subsets. Data are represented as mean ± SEM. (PDF 45kb).
