# Supplementary material for: Identification and characterisation of transient receptor potential melastatin 2 and CD38 channels on natural killer cells using the novel application of flow cytometry
Source: BMC Immunol. 2019 May 10;20:14. doi: 10.1186/s12865-019-0293-0 (PMC6509826; doi:10.1186/s12865-019-0293-0)
Supplement: Supplementary file 25 — Gating strategy for the analysis of TRPM2 and CD38 surface expression on NK subsets using primary TRPM2 antibody (1:10) at 1 h/30 min. (PDF 44 kb) [file 12865_2019_293_MOESM25_ESM.pdf]

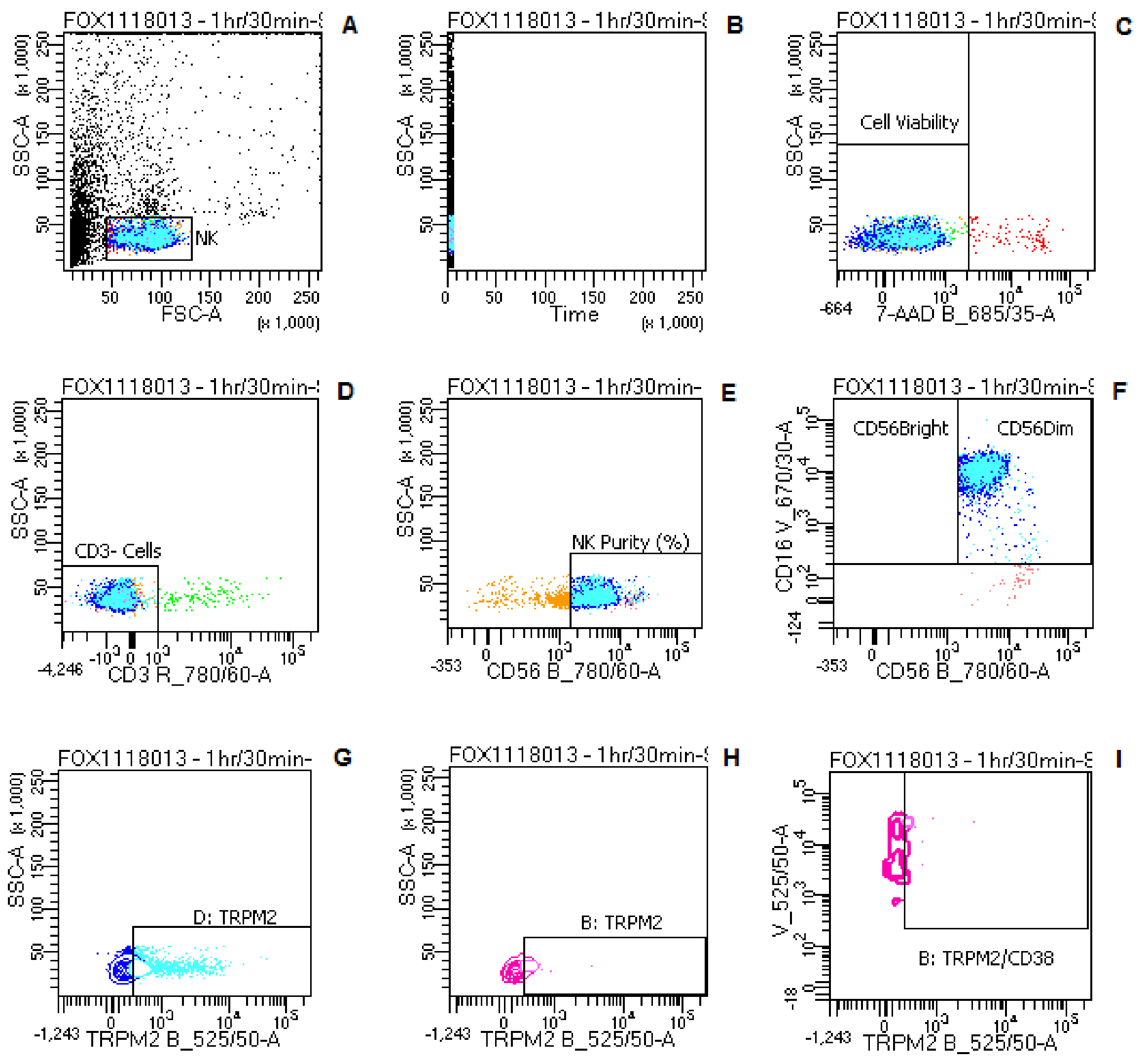

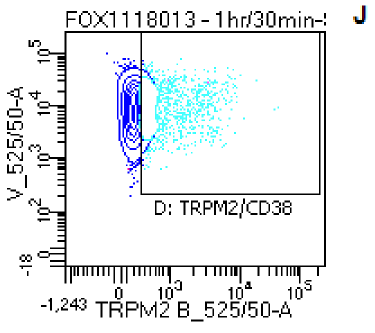

Tube: S1:10

Population	#Events	%Parent	%Total
All Events	10,000	####	100.0
NK	6,276	62.8	62.8
Cell Viability	6,171	98.3	61.7
CD3- Cells	6,031	97.7	60.3
NK Purity (%)	5,566	92.3	55.7
CD56Bright	59	1.1	0.6
B: TRPM2	8	13.6	0.1
B: TRPM2/CD38	10	16.9	0.1
CD56Dim	5,499	98.8	55.0
D: TRPM2	1,003	18.2	10.0
D: TRPM2/CD38	1,149	20.9	11.5

Figure S25. Gating strategy of TRPM2 and CD38 using primary TRPM2 antibody (1:10) at 1hr/30min.

Additional file 25: **Figure S25.** Gating strategy of TRPM2 and CD38 using primary TRPM2 antibody (1:10) at 1hr/30min. **(a)** Lymphocyte populations were identified using forward and side scatter dot plots. (**b)** Duration of cell acquirement was measured followed by **(c)** cell viability after 7-AAD staining. **(d)** $CD3^{+}$ cells were excluded and only **(e)** $CD3^{-}$ lymphocytes were further used to characterise NK cells by CD56. **(f)** $CD3^{-}/CD56^{+}$ NK cells were sorted into $CD56^{Bright}CD16^{Dim/-}$ and $CD56^{Dim}CD16^{/+}$ NK cell subsets using CD56 and CD16. TRPM2 surface expression was measured on **(g)** $CD56^{Dim}CD16^{/+}$ and **(h)** $CD56^{Bright}CD16^{Dim/-}$ NK cell subsets. Dual surface expression of TRPM2 and CD38 was furthermore assessed on **(i)** $CD56^{Bright}CD16^{Dim/-}$ and **(j)** $CD56^{Dim}CD16^{/+}$ NK cell subsets. Data are represented as mean ± SEM. (PDF 45kb).
